# Discrimination tasks in simulated low-dose CT noise

**DOI:** 10.1002/mp.16412

**Published:** 2023-04-14

**Authors:** Craig K. Abbey, Frank W. Samuelson, Rongping Zeng, John M. Boone, Kyle J. Myers, Miguel P. Eckstein

**Affiliations:** 1 Department of Psychological and Brain Sciences, University of California, Santa Barbara, California, USA; 2 Division of Imaging, Diagnostics and Software Reliability, US Food and Drug Administration, Silver Spring, Maryland, USA; 3 Departments of Radiology and Biomedical Engineering, University of California, Davis, California, USA; 4 Puente Solutions, LLC, Phoenix, Arizona, USA

**Keywords:** discrimination tasks, observer performance, ramp-spectrum noise

## Abstract

**Background:**

This study reports the results of a set of discrimination experiments using simulated images that represent the appearance of subtle lesions in low-dose computed tomography (CT) of the lungs. Noise in these images has a characteristic ramp-spectrum before apodization by noise control filters. We consider three specific diagnostic features that determine whether a lesion is considered malignant or benign, two system-resolution levels, and four apodization levels for a total of 24 experimental conditions.

**Purpose:**

The goal of the investigation is to better understand how well human observers perform subtle discrimination tasks like these, and the mechanisms of that performance. We use a forced-choice psychophysical paradigm to estimate observer efficiency and classification images. These measures quantify how effectively subjects can read the images, and how they use images to perform discrimination tasks across the different imaging conditions.

**Materials and Methods:**

The simulated CT images used as stimuli in the psychophysical experiments are generated from high-resolution objects passed through a modulation transfer function (MTF) before down-sampling to the image-pixel grid. Acquisition noise is then added with a ramp noise-power spectrum (NPS), with subsequent smoothing through apodization filters. The features considered are lesion size, indistinct lesion boundary, and a nonuniform lesion interior. System resolution is implemented by an MTF with resolution (10% max.) of 0.47 or 0.58 cyc/mm. Apodization is implemented by a Shepp-Logan filter (Sinc profile) with various cutoffs. Six medically naïve subjects participated in the psychophysical studies, entailing training and testing components for each condition. Training consisted of staircase procedures to find the 80% correct threshold for each subject, and testing involved 2000 psychophysical trials at the threshold value for each subject. Human-observer performance is compared to the Ideal Observer to generate estimates of task efficiency. The significance of imaging factors is assessed using ANOVA. Classification images are used to estimate the linear template weights used by subjects to perform these tasks. Classification-image spectra are used to analyze subject weights in the spatial-frequency domain.

**Results:**

Overall, average observer efficiency is relatively low in these experiments (10%–40%) relative to detection and localization studies reported previously. We find significant effects for feature type and apodization level on observer efficiency. Somewhat surprisingly, system resolution is not a significant factor. Efficiency effects of the different features appear to be well explained by the profile of the linear templates in the classification images. Increasingly strong apodization is found to both increase the classification-image weights and to increase the mean-frequency of the classification-image spectra. A secondary analysis of “Unapodized” classification images shows that this is largely due to observers undoing (inverting) the effects of apodization filters.

**Conclusions:**

These studies demonstrate that human observers can be relatively inefficient at feature-discrimination tasks in ramp-spectrum noise. Observers appear to be adapting to frequency suppression implemented in apodization filters, but there are residual effects that are not explained by spatial weighting patterns. The studies also suggest that the mechanisms for improving performance through the application of noise-control filters may require further investigation.

## 1 | INTRODUCTION

An important set of tasks in medical imaging can be described as lesion-discrimination tasks in which fine features of a readily visible object are used for classification. Assessments of lung nodules in CT images, in which nodules are labeled as malignant or benign, generally fall into this category. While the lung nodules can be readily detected—even relatively low-contrast ground-glass opacities—it is often more difficult to determine their malignant potential on the basis of nodule features. This makes lesion-discrimination tasks challenging, and therefore potentially useful endpoints for evaluations of task-based image quality.^1–5^

In terms of the radiological search literature,^6–12^ lesion-discrimination tasks can impact the role of search in task performance relative to other common radiological tasks such as low-contrast detection. In principle, a clearly visible lesion profile should eliminate errors of search and recognition, defined respectively by a failure to fixate or a short dwell time in eye-tracking studies. This puts the focus on decision processes as the primary source of error (i.e., errors made after a longer dwell time). In practice, search remains an important component of real-world high-contrast tasks in medical imaging because of large 3D search regions with cluttered backgrounds. Nevertheless, eye-tracking studies have also established that decision errors remain a substantial source of error in lung-nodule assessments and other relevant clinical discrimination tasks.^13–15^ This motivates a better understanding of how human observers discriminate between readily visible objects with subtle signs of abnormality, which could help optimize imaging systems, image processing, and the development of decision support for optimal imaging performance.

The approach taken here is based on previous studies that use Gaussian statistical processes to simulate images with specified resolution and noise properties.^16–19^ The simulation includes so-called ramp-spectrum noise found in tomographic imaging in which noise power increases proportionally with spatial frequency^20–22^ up to image apodization filters implemented for noise control. One advantage of the Gaussian-process approach for simple discrimination tasks is that an analytic expression for the likelihood ratio exists, facilitating an Ideal-Observer calculation^23–25^ and leading to an efficiency characterization of human-observer performance. Efficiency analysis quantifies how effectively diagnostic information in images is being used by human observers to perform tasks. It can be factored into terms related to internal noise^26^ and sampling efficiency,^27–29^ a measure of perceptual tuning, which further characterize performance.

While efficiency analysis provides a quantitative measure of how much diagnostic information is being used by an observer, a different psychophysical technique, classification-image analysis, investigates how that information is being accessed.^30^ This approach uses noise-fields in combination with decisions to estimate the spatial weights used by an observer to perform a task. Thus, it gives a sense of how image pixels are converted into a decision about the object being imaged. Classification-image analysis (and reverse-correlation methods more generally^31^) is based on an assumption of Gaussian distributed images.^32–34^ The combination of efficiency analysis and classification-image analysis allow for a more in-depth view of the transfer of information to observers in imaging tasks.

To better understand human-observer performance in this setting, we have evaluated a series of discrimination tasks using simulated images with features that are related to lung-cancer screening. The image stimuli are subject to resolution limitations and masking by ramp-spectrum noise textures with properties that are qualitatively similar to nodules in low-dose CT images of lung parenchyma. We use this approach to evaluate 3 different discrimination tasks (size, edge sharpness, lesion uniformity), 2 imaging systems (low-resolution and high-resolution), and 4 levels of apodization (smoothing) for a total of 24 experimental conditions.

We use two relatively novel approaches to analyzing classification images in this work, in addition to a number of fairly established techniques. The use of “unapodized” classification images has been recently published.^35^ This technique allows us to make more direct comparisons of classification images across apodization conditions. We also introduce the concept of differential sampling efficiency, which allows us to evaluate how well a classification image is tuned for a particular task. Details of these two novel approaches are provided in Sections 2.5.4 and 2.5.2.

## 2 | METHODS

### 2.1 | Discrimination tasks

This work considers discrimination tasks involving three features that are conceptually related to the detection of lung cancer with CT,^36–38^ shown in Table 1. We briefly describe the tasks here, with more formal mathematical descriptions below. The features in each task are taken from clinical features that are known to be relevant to the characterization of lung lesions in CT, but they are simplified and abstracted into mathematical forms that are amenable to experimentation in psychophysical studies. Task 1 is a size-discrimination task, in which a slightly larger lesion is discriminated from a smaller baseline lesion with a 3 mm diameter (FWHM). The task parameter is the difference in radius between the larger and smaller lesion. Task 2 can be described as discriminating edge sharpness between lesions, in which a 5 mm diameter lesion with a more slowly decaying edge (malignant) is discriminated from a similar size lesion with a more distinct edge (benign). In this case the task parameter controls the rate of decay at the edge of the lesion, which represents the profile of poorly circumscribed invasive lesions. Task 3 can be described as a lesion uniformity discrimination task, in which a 5 mm lesion with region of low interior attenuation (malignant) is discriminated from one with a uniform interior (benign). A subtle nonuniform lesion interior can be a sign of malignant processes such as necrosis or local edema.^39^

#### 2.1.1 | Task profiles

**FIGURE 1:**
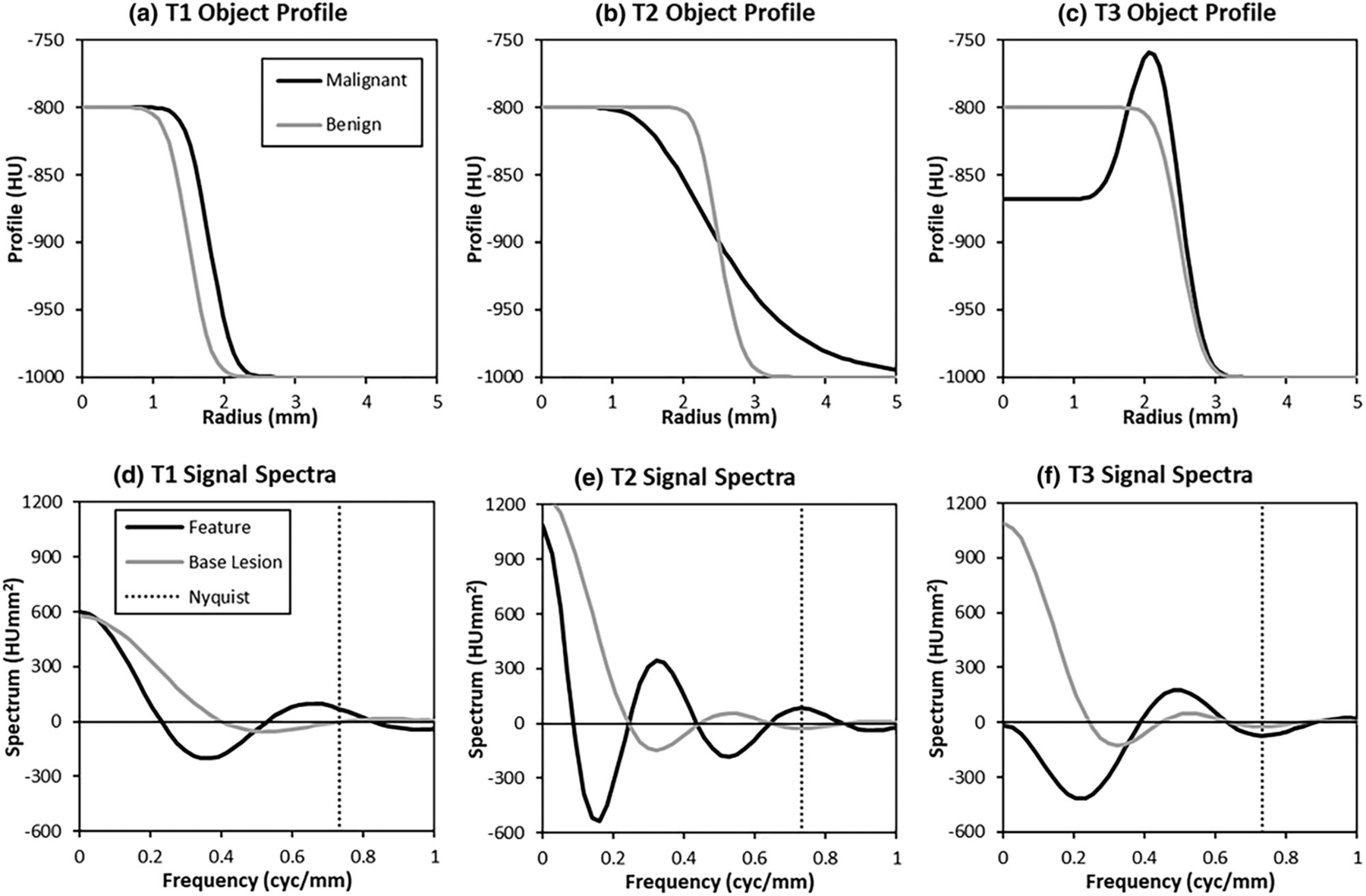
Task profiles. Radial plots of the “Malignant” and “Benign” profiles (a–c) are shown for each of the three tasks considered (T1–T3). In Task 1, the feature of interest is the lesion size. In Task 2, the feature of interest is an indistinct or unsharp boundary. In Task 3, the feature of interest is a nonuniform lesion interior. The spectral plots (real part of the Fourier Transform) for each task (d–f) show that the spectrum of the features falls off more slowly than that of the base lesion used for the task. Thus the feature discrimination tasks tend to place more weight on higher spatial frequencies. Note that the lesion profiles have been scaled to match the integrated spectral power of the features in each task. The legend on the left applies to each row of plots.

Target (malignant) and alternative (benign) signal profiles for each task are plotted in the top row of Figure 1a–c. All lesions used as image stimuli are radially symmetric and therefore can be defined by radial plots. To make the stimuli, target and alternative “objects” based on these radial profiles are generated at high resolution, and then passed through a transfer function and down sampled. The result is embedded in a noise field to produce an image stimulus. The mean lesion intensity, L=200 is defined in Hounsfield Units (HU), with a background intensity of B=−1000HU, the intensity of air. This gives the lesions a nominal intensity of −800 HU, which is consistent with weak ground-glass lesions found clinically.^40^ Let r represent the distance of a point from the center of the image, $r={\left(x^{2}+y^{2}\right)}^{1/2}$, then the radial profiles of the “benign” lesions are given by

(1.1) $$s_{0}\left(x,y\right)=B+L\Phi \left(\frac{R_{L}-r}{\sigma_{L}}\right),$$

where Φ is the cumulative normal distribution function, $R_{L}$ is the radius of the lesion, and $\sigma_{L}$ provides some intrinsic smoothness to the object profile. Task 1 considers size discrimination for small lesions or nodules with a diameter of 3 mm, and therefore $R_{L}=1.5\mathrm{mm}$. For Tasks 2 and 3, a somewhat larger lesion witha5 mm diameter is used as the base, with $R_{L}=2.5\mathrm{mm}$. The $\sigma_{L}$ parameter is set to 0.25 mm for all conditions. In addition to implementing an intrinsic smoothness in the lesions, this parameter also helps to mitigate potential aliasing issues that might appear if a more abrupt Step function were used.

For the size discrimination task, the parameter of interest is the additional size of the “malignant” lesion, ΔR, which leads to the Task-1 malignant signal profile

(1.2) $$s_{T1}\left(x,y;\Delta R\right)=B+L\Phi \left(\frac{R_{L}+\Delta R-r}{\sigma_{L}}\right)$$

For the edge-sharpness task, the parameter of interest is the inflation of the intrinsic smoothness, Δσ, which results in the Task-2 malignant signal profile

(1.3) $$s_{T2}\left(x,y;\Delta \sigma \right)=B+L\Phi \left(\frac{R_{L}-r}{\sigma_{L}+\Delta \sigma }\right).$$

For the lesion-uniformity task, the parameter of interest is the perturbation of the lesion interior, ΔC, which results in the Task-3 malignant signal profile

(1.4) $$s_{T3}\left(x,y;\Delta C\right)=B+L\left(\Phi \left(\frac{R_{L}-r}{\sigma_{L}}\right)+\Delta C\left(\frac{1}{2}\Phi \left(\frac{R_{L}-r}{\sigma_{L}}\right)-\Phi \left(\frac{R_{I}-r}{\sigma_{L}}\right)\right)\right)$$

Note that $R_{l}$ is the radius of the lesion interior, $R_{l}=0.71R_{L}$, and the last term in the equation decreases the intensity of the inner portion of the lesion $\left(r<R_{l}\right)$ by LΔC/2, while it increases the outer portion of the lesion $\left(R_{l}<r<R_{L}\right)$ by the same amount.

Equations (1.1) through (1.4) are used to create object profiles on a finely-sampled grid, as the first step in generating image stimuli. The square sub-region (W=H=87.5mm on each side) is sampled with an isotropic pixel width of 0.076 mm, which represents 9 × oversampling of the final pixel size and an array size of N=M=1152. The sampled x and y are given by $s\left[n,m\right]=s\left(x_{n},y_{m};\theta \right)$ with task parameter defined by the specific equation used to generate the array. All lesions are centered in the sampling grid.

#### 2.1.2 | Object spectra

One notable property of these discrimination tasks is that they tend to emphasize higher spatial frequencies than traditional low-contrast detection tasks. The bottom row of Figure 1d–f shows plots of the Fourier Transform (FT) for the difference signal in each task (T=1−3), $\Delta {\hat{s}}_{T}\left(k,l\right)=FT\left[s_{T}\left(x_{n},y_{m};\theta \right)-s_{0}\left(x_{n},y_{m}\right)\right]$, along with the frequency profile of the corresponding base lesion, the benign profile $\Delta {\hat{s}}_{L}\left(k,l\right)=FT\left[s_{0}\left(x_{n},y_{m}\right)-B\right]$. The caret ^ is used throughout this report to indicate an array that has been transformed to the Fourier domain. The index variables, k and l, represent the indices of the 2D FT. Since these signals are real-valued and rotationally symmetric, the imaginary component of the Fourier Transform is zero. The lesion plots are intended to represent the spectral signal one might use in a lesion-detection task, and the magnitude of L in Equation (1.1) has been scaled in these plots so that the lesion has the same spectral energy as the malignant-benign difference signal for these plots. The plots show that the task spectra fall off somewhat more slowly than the lesion spectra do, which means that there is relatively more spectral energy at higher spatial frequencies for the lesion profile itself.

The decay of spectral energy can be quantified in terms of a “mean frequency” of each spectral profile, defined as

(1.5) $${\mathrm{MF}}_{T}=\frac{\sum_{k=0}^{N-1}\sum_{l=0}^{N-1}f_{k,l}\left|\Delta {\hat{s}}_{T}\left[k,l\right]\right|}{\sum_{k^{\prime }=0}^{N-1}\sum_{l^{\prime }=0}^{N-1}\left|\Delta {\hat{s}}_{T}\left[k^{\prime },l^{\prime }\right]\right|}.$$

The mean frequency of the lesion spectrum is 0.40 cyc/mm for Task 1, and 0.37 for Tasks 2 and 3 (recall that the lesion radius is 1.5 mm for Task 1, and 2.5 mm for Tasks 2 and 3). By contrast, the mean frequency of the feature spectrum is 0.63, 0.55, and 0.50 cyc/mm for Tasks 1–3 respectively, representing a substantial increase (35%–58%) in high-frequency content. The frequency ranges of the task spectra in Figure 1d–f are also seen to extend well beyond the Nyquist frequency of the final image (i.e., before filtering by a system transfer function and down-sampling as seen below) indicating that these tasks are fundamentally limited by the resolution of the imaging systems.

It is also notable that Tasks 1 and 2 retain considerable signal at zero frequency (DC). This means that the integrated malignant lesion profile is larger than the integrated benign lesion profile. As a result, the features in these tasks contain spectral energy that spans the range of frequencies available in the images within the limits of the system MTFs.

### 2.2 | Simulated imaging systems

#### 2.2.1 | System transfer properties

We simulate two imaging systems intended to reconstruct a 35 cm × 35 cm field of view (FOV) with a 512 × 512 array of pixel values (0.68 mm pixel size). The simulation focuses on a 128 × 128 subregion of the FOV. The two imaging systems, referred to as Systems 1 and 2, are defined by different spatial resolution properties. System 1 has a lower resolution than System 2, which is implemented by a system transfer function that falls off more quickly. The modulation transfer functions (MTFs) of both systems are defined by a cosine-rolloff in radial frequency,

(1.6) $$M\left(f\right)=\left\{\begin{array}{ll} \frac{1}{2}+\frac{1}{2}\mathrm{Cos}\left(\frac{\pi f}{f_{0}}\right) & \mathrm{if}f\leq f_{0}\\ 0 & \mathrm{if}f>f_{0} \end{array}\right.$$

with System 1 rolling off to zero at $f_{0}=0.59\mathrm{cyc}/mm$, and System 2 rolling off at $f_{0}=0.73\mathrm{cyc}/mm$ (which is also the Nyquist frequency for the final pixel size of 0.68 mm). This means that System 1 is somewhat oversampled, but this preserves the pixel size and scale of the images for the performance studies.

**FIGURE 2:**
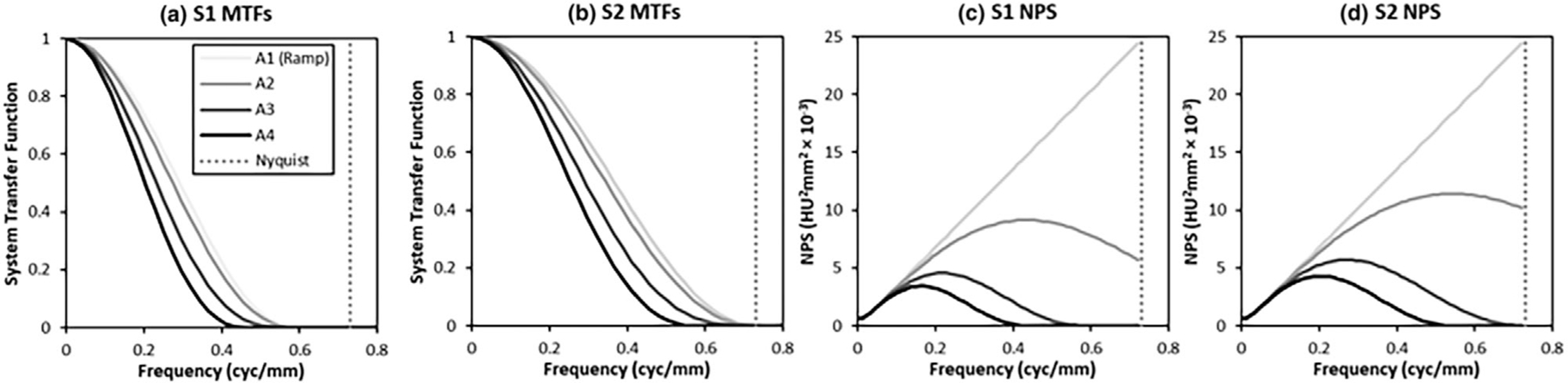
System properties. The simulated modulation transfer functions (a and b) for the low-resolution system (S1) and the high-resolution system (S2) are shown, along with plots of the noise-power spectra (c and d) at each of the 4 apodization levels (A1–A4). The legend on the left applies to all plots.

In addition, both systems employ frequency apodization as a means to control noise. Apodization is implemented by various Sinc-weighted frequency rolloffs, often called a Shepp-Logan filter,^41^ which multiply the MTF according to

(1.7) $$A\left(f\right)=\left\{\begin{array}{ll} \mathrm{Sinc}\left(\frac{\pi f}{f_{c}}\right) & \mathrm{if}f\leq f_{c}\\ 0 & \mathrm{if}f>f_{c} \end{array}\right.,$$

where $f_{c}$ is the cutoff frequency of the apodization filter. The apodization conditions consist of no apodization (A1; $f_{c}=\infty$), a cutoff frequency at 2 times the cutoff of the MTF (A2; $f_{c}=2f_{0}$), a Sinc-rolloff with the first zero at the cutoff of the MTF (A3; $f_{c}=f_{0}$), and a Sinc-rolloff with the first zero at 0.8 times the cutoff of the MTF (A4; $f_{c}=0.8f_{0}$). Figure 2(a and b) shows the resulting combined transfer function $\left(M\left(f\right)\times A\left(f\right)\right)$ of the two systems at each apodization level.

A finely-sampled object profile, $s\left[n,m\right]$, converted into the noiseless mean image by a convolution operation in which the frequency content of the object, $\hat{s}\left[k,l\right]$, is multiplied by a filter, $\hat{h}\left[k,l\right]=A\left(f_{k,l}\right)M\left(f_{k,l}\right)$, before applying an inverse FT. The resulting spatial profile is down-sampled by a factor of 9 in both directions to give the final mean image, $\mu \left[n,m\right]$ with n,m=0,…,127, on a 0.68 mm pixel grid. The task and task-parameters are implicit in the definition of μ. Note that wrap-around effects from the use of finite FTs are expected to be minimal because the lesions are located in the center of the images.

#### 2.2.2 | Noise properties

We simulate noise in the down-sampled images as a ramp-spectrum Gaussian process out to the Nyquist frequency of the images (0.73 cyc/mm). For frequencies less than 5% of Nyquist ($f_{L}=0.0.37\mathrm{cyc}/mm$) the spectrum levels according to a quadratic profile,

(1.8) $$N\left(f\right)=\left\{\begin{array}{ll} \frac{C_{N}}{2}\left(\frac{f^{2}}{f_{L}}+f_{L}\right) & \mathrm{if}f\leq f_{L}\\ C_{N}f & \mathrm{if}f_{L}<f\leq f_{\mathrm{Nyq}} \end{array}\right.,$$

where $C_{N}$ scales the power spectrum to achieve a given noise magnitude. The leveling of the ramp-spectrum at low frequencies is a known component of real systems,^42^ and it also avoids the unrealistic situation in which there are noiseless frequencies that contain signal (i.e., SNR=∞).

**FIGURE 3:**
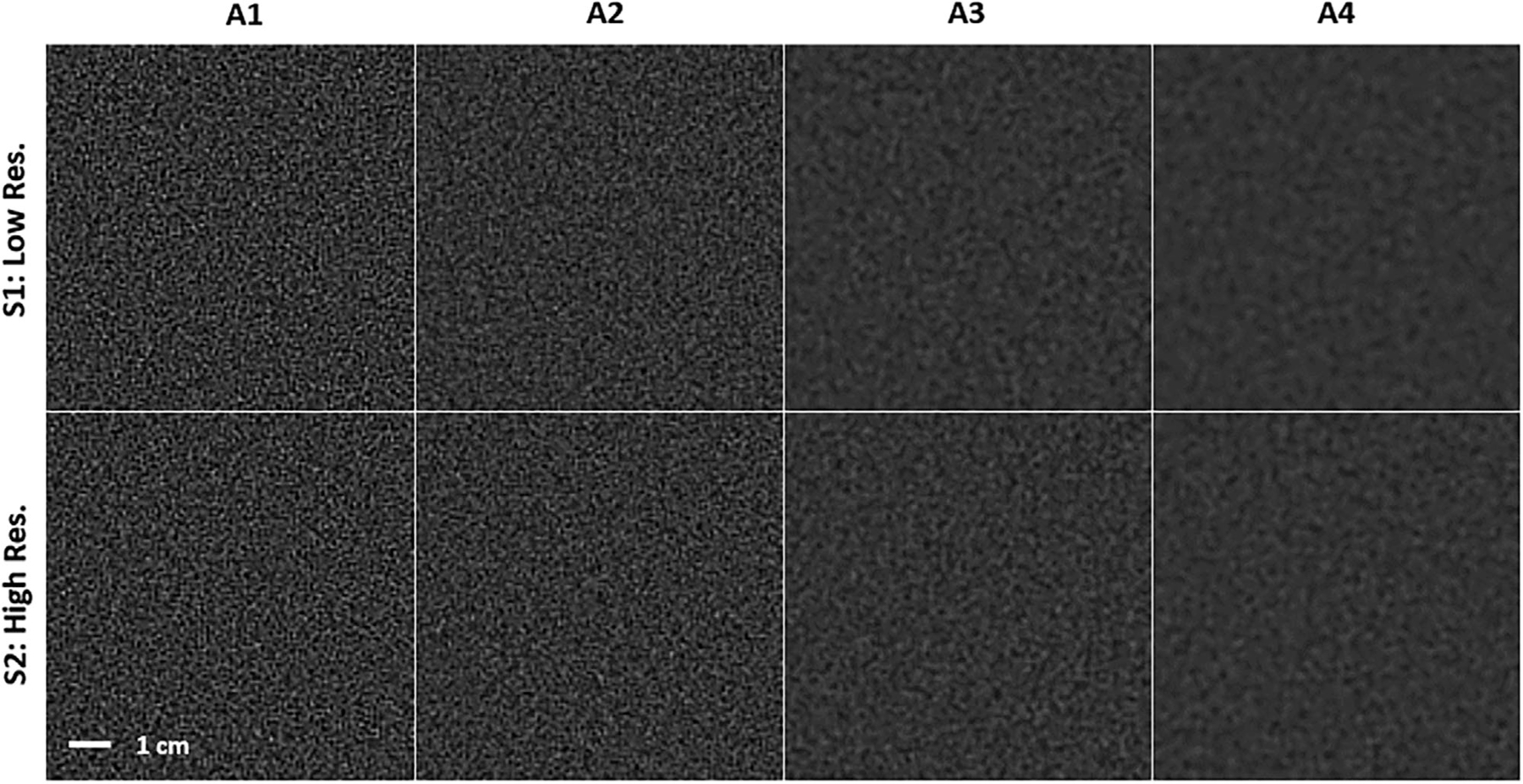
Noise textures. The different levels of apodization lead to different noise amplitude and texture in the simulated imaging systems (S1: Low-Resolution System; S2: High-Resolution System). Higher levels of apodization result in smoother and less grainy texture.

The noise power spectrum is identical for both Systems 1 and 2. In the apodization conditions, noise is attenuated by the frequency rolloff of the various Sinc filters according to the product $N\left(f\right)A{\left(f\right)}^{2}$. Figure 2(c,d) shows the combined NPS (ramp and apodization) of the systems in Hounsfield units (HU) times mm^2^. Figure 3 shows sample noise textures for each system and apodization level.

Noise samples are generated by filtering white noise. Let $\hat{z}\left(k,l\right)$ represent a sample of standardized Gaussian white noise that has been transformed to the FT domain. The apodized ramp-spectrum noise sample is computed as the product of the white noise, the apodization spectrum, and the square root of the noise power spectrum, $\hat{n}\left(k,l\right)=\hat{z}\left(k,l\right)N{\left(f_{k,l}\right)}^{1/2}A\left(f_{k,l}\right)$, followed by an inverse FT.

The resulting noise field has a discrete power spectrum given by

(1.9) $$\hat{\sum }\left[k,l\right]=N\left(u_{k},v_{l}\right){\left|A\left(u_{k},v_{l}\right)\right|}^{2}.$$

The result is added to the down-sampled mean image to get a sample stimulus

(1.10) $$g\left[n,m\right]=\mu \left[n,m\right]+n\left[n,m\right].$$

#### 2.2.3 | Image display

**FIGURE 4:**
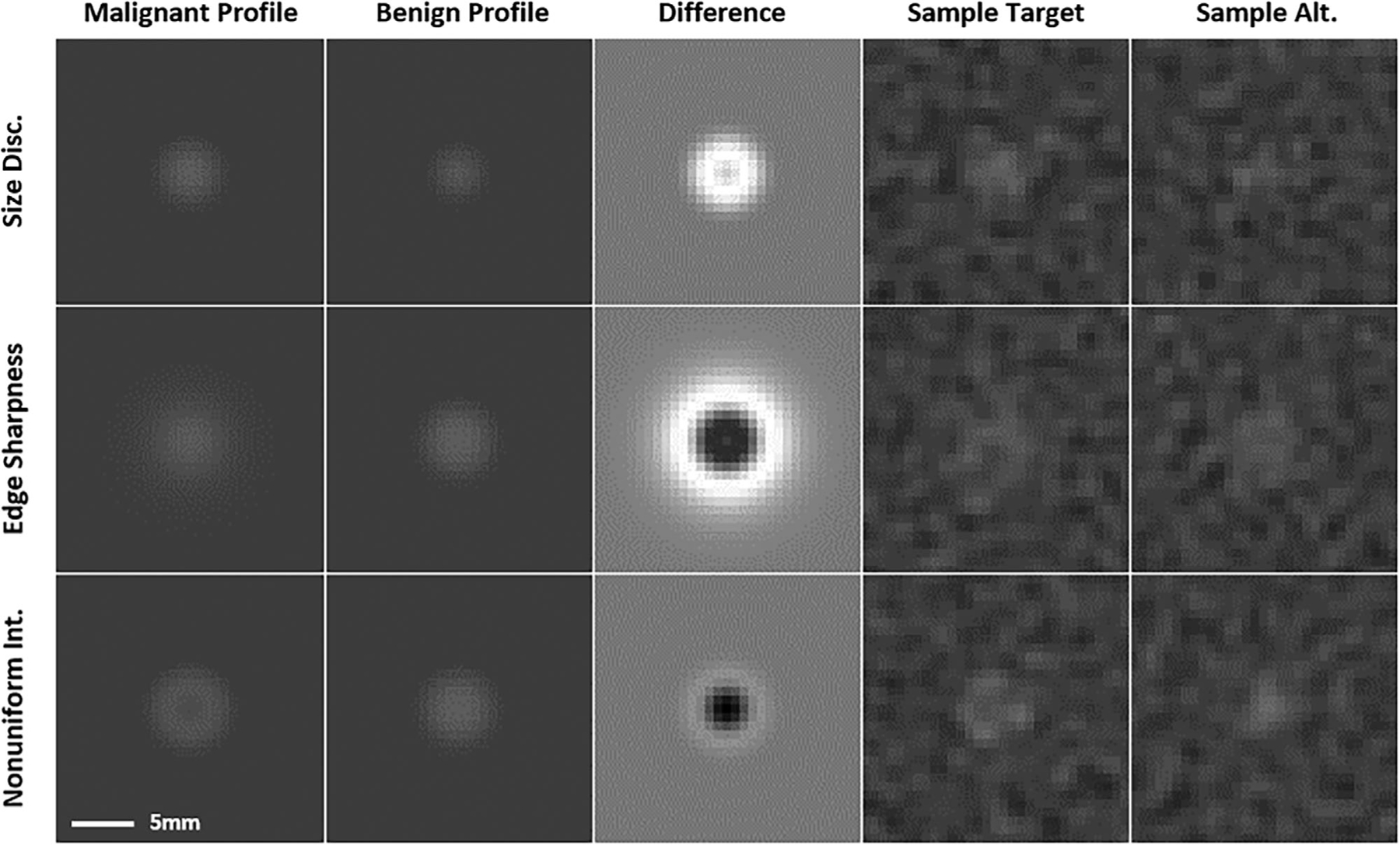
Stimulus profiles and images. The (noiseless) malignant and benign profiles for each of the three tasks are shown (Left side, rows 1–3), along with the difference signal and sample image patches from each class (target and alternative). All patches derived from the low-resolution system, at apodization-level 3. Task parameters have been exaggerated for the purpose of display in this figure. The image patches are cropped from a simulation ROI of 87.5 mm in an assumed 350 mm field of view. All the images have a window of 1500 HU and level of −650 HU except for the difference images (central column) which are scaled to the maximum difference value.

Table 2 gives generic measures of resolution and noise for the two systems and the four apodization conditions. In terms of lung imaging, these values are roughly consistent with the measured values of Kim et al.^43^ for low dose scanning (${\mathrm{CTDI}}_{Vol}=0.38mGy$). Note that the more highly apodized conditions for System 2 overlap with the less apodized conditions of System 1. Image stimuli are scaled for display based on the assumed use of a lung window of 1500 HU and level of −650 HU. Values outside the window are truncated to the window boundaries. After scaling, the window is discretized to 8 bits for display. Figure 4 shows examples of the “signal” (malignant) and “alternative” (benign) profiles along with the difference signal between these two profiles, as well as examples of noisy images.

### 2.3 | Psychophysical procedure

The image simulation procedure is used to generate stimuli for two-alternative forced-choice experiments, where a stimulus from the malignant class and an independent stimulus from the benign class are displayed side-by-side on each trial. The position of the two images (right or left) is randomized on each trial, and the reader is asked to indicate the alternative corresponding to a malignant image using a mouse click. The monitor used for the studies (MD1119; Barco, GA) has a measured luminance range from 0.1 to 162.9 Cd/m^2^, and is calibrated to the DICOM standard. Displayed images have a length and width of 84.5 mm on the display, which represents up-sampling the pixel array by a factor of 2. At a comfortable viewing distance of 75 cm, the Nyquist frequency of the images is 9.9 cycles per degree visual angle, which is well within the typical resolution of the human eye (∼60 cycles/degree).

All observer data was collected under an IRB-approved human-subjects protocol. Each of the 3 tasks had a total of 8 conditions (2 systems and 4 levels of apodization). Subjects completed all the conditions in Task 1, before moving on to Task 2 and then Task 3. Within each task, the 8 conditions were completed in a randomized order. For each subject, every experimental condition began with 6 runs through a staircase training procedure^44^ in which the task parameter is decreased by 15% whenever three correct answers were given in a row, or increased by 15% whenever an incorrect answer was given (a 3-down, 1-up staircase). These runs familiarized the readers with the task, and also allowed us to estimate the signal parameters needed to achieve approximately 80% correct responses for each reader (from the last 5 runs). Subsequently, 2000 trials (40 sessions of 50 trials) were run at the estimated contrast level to estimate the primary endpoints of the study.

Occasionally, a subject performs poorly in the staircase component of the procedure, resulting in a high threshold-contrast estimate that led to substantially higher performance in the classification-image trials. The estimation of classification images has been shown to be weaker as performance increases.^33^ To mitigate these high-performance effects, we repeated experimental conditions—both training and testing—for subjects that achieve more than 90% correct in the classification image trials.

### 2.4 | Task performance analysis

#### 2.4.1 | Contrast energy

The proportion of correct responses (PC) is the natural measure of task performance for 2AFC experiments. However, in these experiments the task parameter has been adjusted, based on the training data, to achieve a PC of approximately 80%, which means that better performance may be reflected in a smaller task parameter. As a result, we use contrast energy to characterize observer performance on each condition. For a given experimental condition, let $\mu_{T}[n,m]$ be the mean pixel-values for the target (malignant) images for a given condition, and let $\mu_{A}[n,m]$ be the column vector representing the alternative (benign) images. The $\mu_{T}$ image will be dependent on the parameter values determined by the staircase procedures for each subject. Contrast energy is defined as

(1.11) $$E_{c}=A_{\mathrm{Pix}}\sum_{n=0}^{127}\sum_{m=0}^{127}{\left\|\mu_{T}[n,m]-\mu_{A}[n,m]\right\|}^{2},$$

where $A_{\mathrm{Pix}}$ is the area of a pixel. Given that the mean images are specified in units of HU, the contrast energy is given in units of HU^2^mm^2^.

Since the proportion correct in our experiments may vary somewhat from the targeted level of 80% correct, we apply a correction to the energy threshold in Equation (1.11) based on the observed PC in the psychophysical experiments. We used the concept of detectability derived from PC,^45^ $d=\sqrt{2}\Phi^{-1}\left(\mathrm{PC}\right)$, with an adjustment based on the detectability of the target PC (d=1.19 for PC = 80%) relative to the observed PC for that condition to obtain the corrected threshold energy,

(1.12) $$E_{c}^{\mathrm{Corrected}}={\left(\frac{d_{\mathrm{Targ}}}{d_{A,c}^{\mathrm{Observed}}}\right)}^{2}E_{c}.$$

Note that if the observed PC is greater than the target PC, then the correction factor will adjust for this by reducing the threshold energy, and vice-versa.

#### 2.4.2 | Performance of the ideal observer

The Ideal Observer (IO) is defined as the optimal classifier for a given task. In two-class discrimination tasks of the sort considered here, with Gaussian-distributed classes and a common noise power spectrum, the IO can be implemented as a weighted sum of pixel values,^25^

(1.13) $$r_{\mathrm{IO}}=\sum_{n=0}^{127}\sum_{m=0}^{127}w_{\mathrm{IO}}[n,m]g[n,m].$$

The weights are defined in the Fourier domain by mean signal profiles and the noise power spectrum as

(1.14) $${\hat{w}}_{\mathrm{IO}}[k,l]=\frac{{\hat{\mu }}_{T}[k,l]-{\hat{\mu }}_{A}[k,l]}{\hat{\sum }[k,l]+\sigma_{Q}^{2}},$$

where the additional term in the denominator represents the additional variance of so-called “quantization” noise.^46^ This term is modeled as having a variance of ΔQ/12, where ΔQ is the quantization step (1500/256 here), and it has the additional effect of stabilizing the computation of ${\hat{w}}_{\mathrm{IO}}$in Equation (1.14) for frequencies where the noise power spectrum is zero from apodization.

The signal-to-noise ratio (SNR) of the ideal observer is defined as the difference in the mean response of the IO to target-present and target-absent images, divided by the response standard deviation. The difference in the mean responses is given by

(1.15) $$\Delta \rho_{\mathrm{IO}}=\sum_{n=0}^{127}\sum_{m=0}^{127}w_{\mathrm{IO}}[n,m]\left(\mu_{T}[n,m]-\mu_{A}[n,m]\right).$$

The standard deviation can be computed in various ways. Our approach is to first define an array, y[n,m], as the product of the noise covariance matrix and the IO template. In the Fourier domain this product is given by

(1.16) $$\hat{y}[k,l]=\left({\hat{\sum }}_{n}[k,l]+\sigma_{Q}^{2}\right){\hat{w}}_{\mathrm{IO}}[k,l].$$

The standard deviation of the ideal observer response is

(1.17) $$\sigma_{\mathrm{IO}}={\left(\sum_{n=0}^{127}\sum_{m=0}^{127}w_{\mathrm{IO}}[n,m]y[n,m]\right)}^{1/2},$$

and the SNR of the Ideal Observer is then given by

(1.18) $${\mathrm{SNR}}_{\mathrm{IO}}=\frac{\Delta \rho_{\mathrm{IO}}}{\sigma_{\mathrm{IO}}}.$$

Since the SNR is dependent on the mean images and the noise covariance, it is affected by the task, the system and the level of apodization that define each experimental condition. In addition, it will change depending on the setting of the task parameter within each condition. As a result, we will consider performance of the IO in relation to performance of each human observer separately.

#### 2.4.3 | Observer efficiency

Task efficiency with respect to the Ideal Observer is considered a measure of the fraction of task-relevant information that is being accessed by an observer. It is defined as the squared ratio of the Human observer SNR relative to ${\mathrm{SNR}}_{\mathrm{IO}}$, as defined above in Equation (1.18). Human observer SNR can be computed directly from proportion correct in 2AFC tasks, and is typically referred to as the detectability index,

(1.19) $$d_{H}=\sqrt{2}\Phi^{-1}\left({\mathrm{PC}}_{H}\right).$$

Efficiency with respect to the IO is then given by

(1.20) $$\eta ={\left(\frac{d_{H}}{{\mathrm{SNR}}_{\mathrm{IO}}}\right)}^{2}.$$

Efficiency is computed for each subject in each experimental condition, and then averaged across subjects to get a final estimate of ensemble performance for the condition.

As we shall see in the results section, it is often the case that human-observer PC deviates somewhat from the targeted value of 80% correct determined from the staircase runs. However, the efficiency estimate in Equation (1.20) is valid even when ${\mathrm{PC}}_{H}$ is different from the targeted value. A higher value of ${\mathrm{PC}}_{H}$ results in a higher value of $d_{H}$ (and vice-versa), but since ${\mathrm{SNR}}_{\mathrm{IO}}$ is computed for the same task parameter, the resulting efficiency represents efficiency at a slightly higher performance threshold. We expect efficiency to be relatively unchanging over small changes in threshold PC, so the observed efficiency values should be representative of efficiency at 80% correct.

#### 2.4.4 | Inference on performance measures

For the purpose of evaluating the significance of the threshold energy and efficiency performance endpoints, we use 3-way ANOVA modeling of log-threshold energy and log-efficiency with Task, System, and Apodization Level as fixed effects that are the focus of the analysis. However the data is subject to variability from the limited set of subjects and cases used in the experiments. Subjects are modeled as a random effect, and cases are aggregated into sessions, which are modeled as a random effect. In the log-threshold energy data, the sessions are the five runs of the staircase procedure that are used to estimate the threshold. For the efficiency analysis, a session is defined as 200 consecutive trials, with a total of 10 sessions for each experimental condition. Models are fit to log-threshold energy and log-efficiency endpoints separately, with main effects, 2-way interactions, and 3-way interactions evaluated for the three fixed effects. The model includes main effects of subjects and sessions as well as all 2-way interactions of these random effects with the fixed effects and each other. The ANOVA uses a Satterthwaite approximation^47^ for degrees of freedom to account for the random effects in the model.

The endpoints of each model are evaluations of the significance of the fixed effect main effects, (3 comparisons), their 2-way interactions (3 comparisons) and 3-way interactions (1 comparison). The two models produce a total of 14 comparisons, with multiple comparisons across both endpoints controlled using the false-discovery rate correction developed by Benjamini et al.^48^ We report false-discovery-rate corrected p-values.

### 2.5 | Classification-image analysis

The classification-image methodology is based on standard methodology for 2AFC studies,^30,32,49^ which uses weighted sums of noise fields that have been filtered by the inverse of the noise covariance matrix. Let $n_{c,j}^{+}\left[n,m\right]$ be the noise field for the target-present stimulus in trial j (for j=1,…,J with J=2000) and experimental condition c (for c=1,…,24), and let $n_{c,j}^{-}\left[n,m\right]$ be the target-absent noise field. Also let $O_{c,i,j}$ be the trial outcome for a given reader (i=1,…,I), defined as 0 or 1 depending on whether the reader responds incorrectly or correctly in the trial. The score-weighted filtered-noise field that serves as the basis for the classification image methodology in 2AFC experiments is defined in the frequency domain as

(1.21) $$\Delta {\hat{q}}_{c,i,j}\left[k,l\right]=\left(O_{c,i,j}-PC_{c,i}\right)\frac{J}{J-1}\frac{{\hat{n}}_{c,j}^{+}\left[k,l\right]-{\hat{n}}_{c,j}^{-}\left[k,l\right]}{{\hat{\sum }}_{c}\left[k,l\right]+\sigma_{Q}^{2}},$$

where $PC_{c,i}$ is the estimated proportion correct for reader i in condition c. The $j/\left(j-1\right)$ term reflects the use of a sample estimate of PC.^33^ While Equation (1.21) defines the score-weighted noise field in the frequency domain, this is typically transformed back to the spatial domain for display and other purposes. Under the assumption of a linear decision variable (i.e., a weighed sum of pixel values), the expectation of Δq is directly related to the pixel weights used to perform the task.^32,33^ We estimate these weights for a given subject by averaging over the trials. We can then estimate the average weights across subjects to reduce the estimation error and evaluate spatial weighting at the group level,

(1.22) $$w_{c}[n,m]=\frac{1}{IJ}\sum_{i=1}^{I}\sum_{j=1}^{J}\Delta q_{c,i,j}[n,m].$$

From this estimate (in units of HU^−1^), we can also obtain an estimate of frequency weights by taking the Fourier Transform of $w_{c}[n,m]$.

Despite averaging across 2000 trials and data from multiple observers, classification images are still subject to sampling error. This error can be large, particularly when there are small values in the dominator of Equation (1.21) due to apodization or simply from low noise levels. To mitigate this error, we apply various smoothing approaches including spatial windowing, frequency windowing, and radial smoothing. Spatial and frequency windowing are implemented using 4^th^-order Butterworth filters. The spatial window has a radius (half -max) of 7.5 mm, which extends considerably past the extent of the objects as shown in Figure 1. The frequency window has a radius of 0.4 cyc/mm, which makes it extend further into the frequency domain than the system-transfer functions in Figure 2. The frequency windowing procedure also zeros the imaginary component of the FT, thereby enforcing symmetry about the midpoint of the classification image. Radial smoothing consists of averaging the spatial classification image across radial bands in the spatial domain.

#### 2.5.1 | Classification-image spectra and spectral features

Classification-image spectra are defined by the Fourier transform of $w_{c}$ (with spatial windowing and radial smoothing), after shifting so that the central pixel of the image is at the origin of the transform and multiplying by the pixel area (so that they have units of HU^−1^mm^2^). Since the classification images are approximately rotationally symmetric, we compute 1-dimensional spectral plots by averaging the frequency components in radial bins with a width of a frequency sample, $\Delta_{Freq}=1/N\Delta_{Pix}$. Note that any imaginary part of the spectrum will average to zero over these symmetric regions.

We use features of the classification image spectrum to quantify the effects of different imaging conditions on the average classification weights. Let ${\hat{w}}_{c}\left[k,l\right]$ represent the FT of the classification image defined in Equation (1.22). We choose two such features that integrate spectral power over a limited frequency range. The first is the integrated power of the spectrum

(1.23) $${\mathrm{IP}}_{c}=\Delta_{\mathrm{Freq}}^{2}\sum_{f_{k,l}<0.35}{\left|{\hat{w}}_{c}[k,l]\right|}^{2},$$

where $f_{k,l}$ is the radial frequency associated with the [k,l] frequency indices ($f_{k,l}=\sqrt{u_{k}^{2}+v_{l}^{2}}$) and the sum considers only indices for which $f_{k,l}\leq 0.35$. The restriction on the frequency range is chosen because the high apodization conditions (levels 3 and 4) tend to get unstable above this frequency level. An increase in the spectral power across conditions occurs when the magnitude of weights increase within the frequency range of the feature. This can occur when internal noise is reduced, since internal noise serves to down-weight the classification image.^32^

The second feature is the mean frequency of the spectral power, defined as

(1.24) $${\mathrm{MF}}_{c}=\frac{\Delta_{\mathrm{Freq}}^{2}\sum_{f_{k,l}<0.35}f_{k,l}{\left|{\hat{w}}_{c}[k,l]\right|}^{2}}{IP_{c}},$$

which is effectively the balance point of the classification image radial power spectrum. This feature is given in frequency units (mm^−1^) and quantifies the distribution of spectral weights in terms of higher or lower frequencies.

Similar to Section 2.4.4, three-way ANOVA models are used to establish the significance of effects for these spectral features. Task, system resolution, and apodization level are the three fixed effects considered, and main effects and two-way interactions are evaluated. Subjects are considered a random effect, but session effects are not modeled since instability makes some classification images difficult to estimate within a session. Integrated power and mean frequency are modeled separately, with multiple comparisons for the two features (12 total) controlled using a 5% false-discovery rate correction. We report corrected *p*-values.

#### 2.5.2 | Sampling efficiency from classification images

An efficiency value, as defined in Equation (1.20), of less than 100% reflects suboptimal performance. This inefficiency may arise from multiple sources, including poor tuning of a discrimination filter and internal noise. The concept of Sampling Efficiency focuses on systematic effects that result from suboptimal tuning of the linear weights. Originally estimated from the slope of threshold energy plotted against noise spectral density,^27,29,50–52^ more recent approaches^53,54^ have used classification images as a way to estimate sampling efficiency.

For a linear discrimination template, w[n,m], the sampling efficiency is computed by first computing a template signal-to-noise ratio, ${\mathrm{SNR}}_{w}$ that is identical to ${\mathrm{SNR}}_{\mathrm{IO}}$ in Equation (1.18), except that the estimated classification image, w[n,m], is used in place of $w_{\mathrm{IO}}[n,m]$. The sampling efficiency is then given by

(1.25) $$\eta_{w}^{\mathrm{Samp}}={\left(\frac{{\mathrm{SNR}}_{w}}{{\mathrm{SNR}}_{IO}}\right)}^{2}.$$

It is clear from the equation that $\eta_{w}^{\text{Samp}}=100\%$, if $w[n,m]=w_{\mathrm{IO}}[n,m]$. It is possible to achieve a sampling efficiency of 100% even if total efficiency, as defined in Equation (1.20), is considerably less than 100% because of internal noise. Thus, sampling efficiency can be thought of as a way to decompose efficiency into a filter tuning component and a residual efficiency component representing effects of internal noise. However, estimation error in the classification image can have a substantial effect on ${\mathrm{SNR}}_{w}$, generally biasing it to be too low. As a result, relatively aggressive smoothing is used to control noise as described above (spatial and frequency windowing, and radial averaging) involving both the spatial and frequency windows described above.

#### 2.5.3 | Differential sampling efficiency

Low sampling efficiency can be interpreted as evidence of poor tuning of subjects’ spatial weighting for a given task. This implies that some components of the weighing profile may be too large, and others too small, and it is of interest to identify these components. To do this, we introduce the concept of differential sampling efficiency, which consists of quantifying the effects of small perturbations of the classification image on sampling efficiency. We will focus on frequency perturbations here. Let ${\hat{w}}_{c}$ be the classification image in the frequency domain, and we define the perturbed classification as ${\hat{w}}_{c}+\varepsilon \delta_{k,l}$, where $\delta_{k,l}$ is a Kronecker Delta (zero everywhere except at index $\left[k,l\right]$, where it is 1) and ε is a small positive constant with the units of $\hat{w}$ (HU^−1^mm^2^). The differential sampling efficiency at this frequency index is then given by

(1.26) $$\Delta \eta_{w}^{\mathrm{Samp}}\left[k,l\right]=\underset{\varepsilon \to 0}{\mathrm{Lim}}\frac{\eta_{w+\varepsilon \delta_{k,l}}^{\mathrm{Samp}}-\eta_{w}^{\mathrm{Samp}}}{\varepsilon }$$

This is equivalent to taking the gradient of sampling efficiency with respect to each frequency in the classification image. Note that any imaginary components of the perturbation are zeroed in the spatial domain forcing the perturbation to be symmetric, and the spatial window is applied to the perturbation as well. In practice we choose a small value for ε by finding a value that has less than 1% effect on the standard deviation of the template.

When the differential sampling efficiency is positive, it means that the sampling efficiency of the weighting pattern is increased by increasing the $\left[k,l\right]$ component. When the component being evaluated has a positive value, this indicates that the component is underweighted. If the component being evaluated is negative, it indicates overweighting. Conversely, when differential sampling efficiency is negative, the sampling efficiency of the weighting pattern is decreased by increasing this component, indicating overweighting or underweighting depending on the sign of the component. The differential sampling efficiency gives us a way to assess the tuning of spatial weighting patterns on a frequency specific basis. We find it convenient to display these after radial averaging as is done for the classification image spectra.

#### 2.5.4 | “Unapodized” classification images

One of the goals of this work is a better understanding of how apodization impacts performance in these sorts of discrimination tasks. However, classification images may be somewhat difficult to compare directly across apodization conditions because the signal and noise properties of the stimuli change in each condition. As a result, it is not clear whether the differences in classification images across apodization conditions represent an appropriate response to different image statistics or some sort of perceptual difference. To put this a different way, an Ideal Observer would also change its spatial weighting across the different apodization conditions (because of the different signal and noise properties), so differences in the classification-image spectra may be ambiguous.

To resolve this ambiguity, we evaluate so-called “unapodized” classification images, described previously.^35^ To estimate these, we use Equation (1.21) with subject responses from the experiments with apodized images, but we generate the classification images (and their spatial frequency spectra) using the unapodized noise fields, defined as Apodization Level 1. The effect of this alteration of the standard classification-image procedure is to treat apodization as if it were part of the perceptual process of the observer rather than an image processing step. The unapodized classification images allow for a direct comparison of classification images across different apodization conditions (for a given task and imaging system), and they also allow for comparison with the ideal observer. Unapodized classification images for the Ideal Observer are invariant across apodization conditions, up to apodization filters that are not invertible. In this case, the IO classification images are invariant for frequencies not in the null-space of the apodization operator.

Once the unapodized classification images have been estimated, we can use the methods described above to display their radial spectra and make inferences on the feature values.

## 3 | RESULTS

A total of six subjects completed the experiments reported here (including 1 co-author of this work and 5 subjects naïve to the goals of the research). The primary findings of the study are described below, including the performance results, the classification images, and classification image spectra.

### 3.1 | Task performance

**FIGURE 5:**
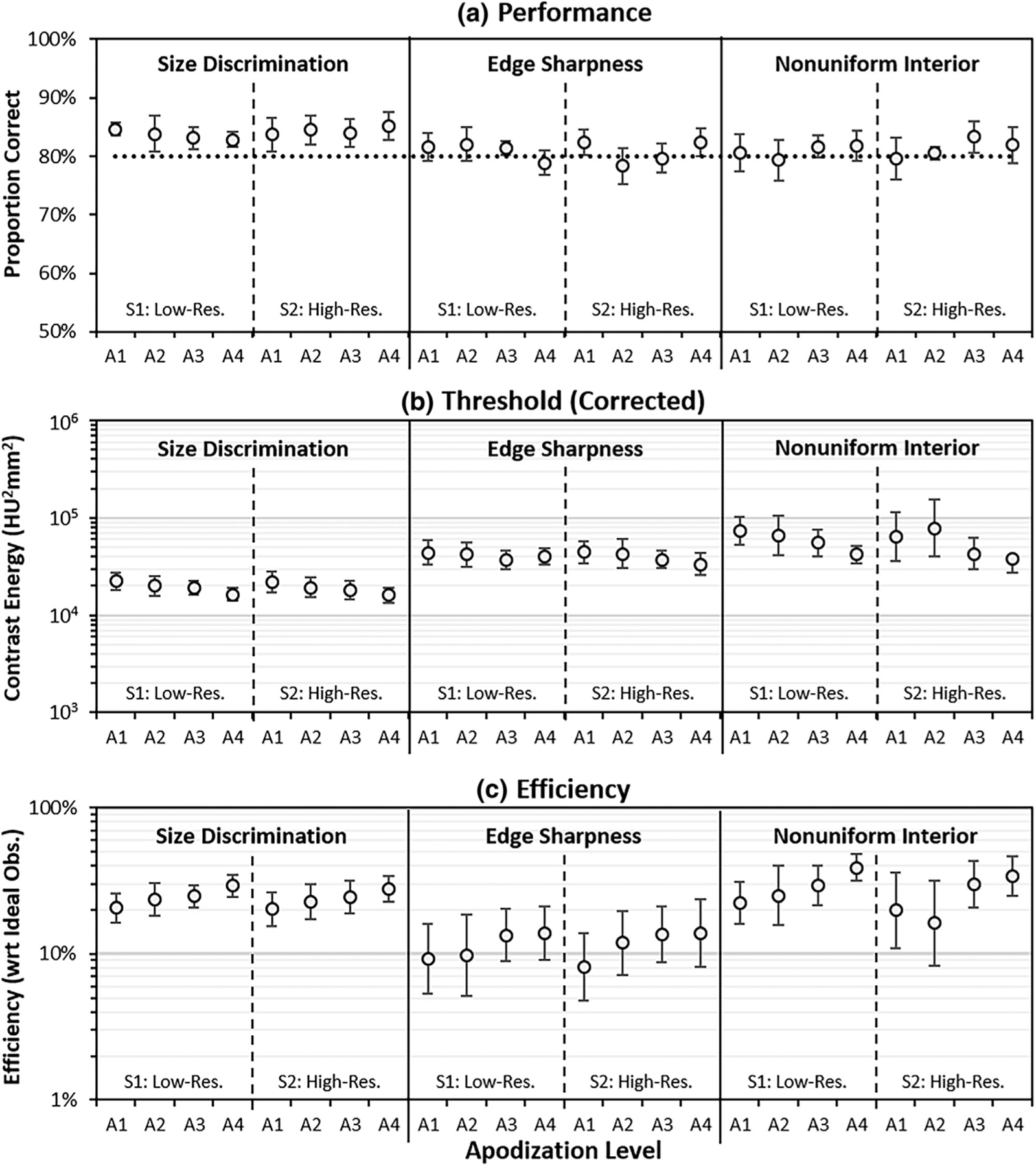
Characterization of performance. The plots show performance for the three tasks, both imaging systems (S1, S2), and the four levels of apodization (A1–A4). The PC plot (A) shows some deviation from the target PC of 80%. The corrected threshold energy (B) and efficiency (C) plots show variability between the tasks and evidence of better performance with increasing apodization (see text). Each estimate is the average performance across the 6 subjects in the studies, with error bars representing ± 1.96 standard errors of the mean.

Three measures of task performance are plotted for each experimental condition in Figure 5. The observed values of average proportion correct (Figure 5a) exhibit some relatively small deviations from the targeted value of 80%, with an apparent bias towards higher values across the conditions. This likely reflects some additional task-specific learning by readers over the course of the 2000 experimental trials that followed the initial threshold estimate. The values are all well within ±10% of the targeted level, although it should be noted that one subject repeated three conditions due to high performance (>90%, see Section 2.3). In all three cases, repeating the condition resulted in an acceptable observed PC.

The middle panel in Figure 5b shows the PC-corrected threshold contrast for each condition, corrected from the observed PC to 80% correct. The observed contrast energy values range over two orders of magnitude from 1.6 × 10^4^ to more than 1.6 × 10^6^. It is clear that Task 3 has substantially higher threshold contrast energy than the other tasks. Within a given task and imaging system, there is evidence of a downward trend in SNR with increasing apodization level. The threshold contrast energy decreases by an average of 22% going from no apodization (A1) to the maximal apodization (A4). This indicates that performance within a task seems to be improving with more apodization.

The bottom panel in Figure 5c shows reader efficiency in each condition. Average efficiency for each task is 25%, 14%, and 30% in Tasks 1–3, respectively. It is notable that Task 3 results in the highest observed efficiency. This shows that the relatively high thresholds in Figure 5B represent the intrinsic difficulty of Task 3 relative to the other tasks, instead of some limitation in the subjects. The efficiency results also show an apparent increase in reader efficiency with increasing levels of apodization. On average, efficiency is 51% higher at full apodization (A4) relative to no apodization (A1). This indicates that the apodization-related improvements seen in contrast energy thresholds represent more effective reading for higher levels of apodization.

As described in Section 2.4.4, mixed-effect linear statistical models and 3-way analysis of variance (ANOVA) are used to assess the significance of trends in log-contrast-threshold energy and log-efficiency plotted in Figure 5, with task, imaging system, and apodization as the fixed features of analysis. We find significant main effects for task and apodization for both endpoints (FDR-corrected *p* < 0.002 in all four cases). In the log-contrast-threshold energy data we find a significant 3-way interaction (FDR-corrected *p* < 0.007), suggesting that different tasks and systems may require different levels of optimization. In the log-efficiency data we find significant interactions between task and apodization, task and imaging system, and a 3-way interaction between the factors (FDR-corrected *p* < 0.0001 in all three cases). These multiple interactions show that effective reading of images can be dependent on multiple factors.

### 3.2 | Classification images

**FIGURE 6:**
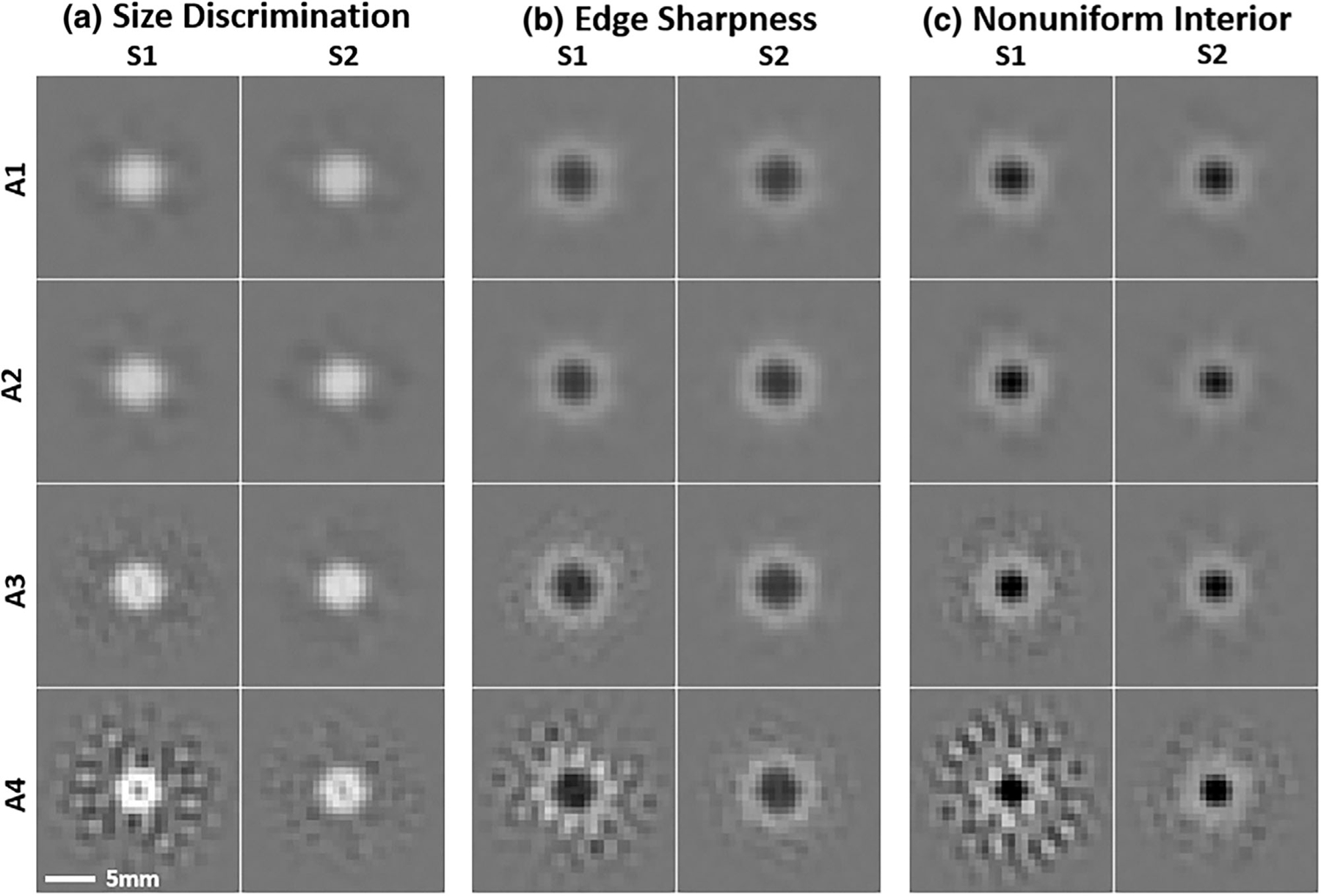
Classification Images. The average classification image is shown for each task, system (S1: Low-Resolution, S2: High-Resolution), and apodization level (A1–A4). These patches are cropped for display purposes and have been spatially windowed to a radius of 7.5 mm (HWHM), and frequency windowed to 0.4cyc/mm. Within each Task (a–c), the display range is held fixed. At the higher levels of apodization, instability in the estimation process is evident.

Classification images, averaged across subjects according to Equation (1.22) for each task, are shown in Figure 6 after spatial windowing and smoothing with 4th-order Butterworth filters as described in Section 2.5. The classification images generally show clear regions of facilitation (positive weighting) and inhibition (negative weighting). The conditions with greater levels of apodization (bottom of Figure 6) appear to have more variability (estimation error) than the others. This is a consequence of instability due to the inverse covariance matrix applied in the classification estimation procedure. There are also substantial differences between the classification images for the different tasks. In Task 1, the classification images are generally facilitatory near the lesion boundary, with a mild inhibitory region outside of this area. The central region of the classification image appears to be suppressed at higher levels of apodization. In Task 2, there is a pronounced inhibitory central region with a facilitatory surround, and this pattern persists in Task 3.

### 3.3 | Classification image spectra

**FIGURE 7:**
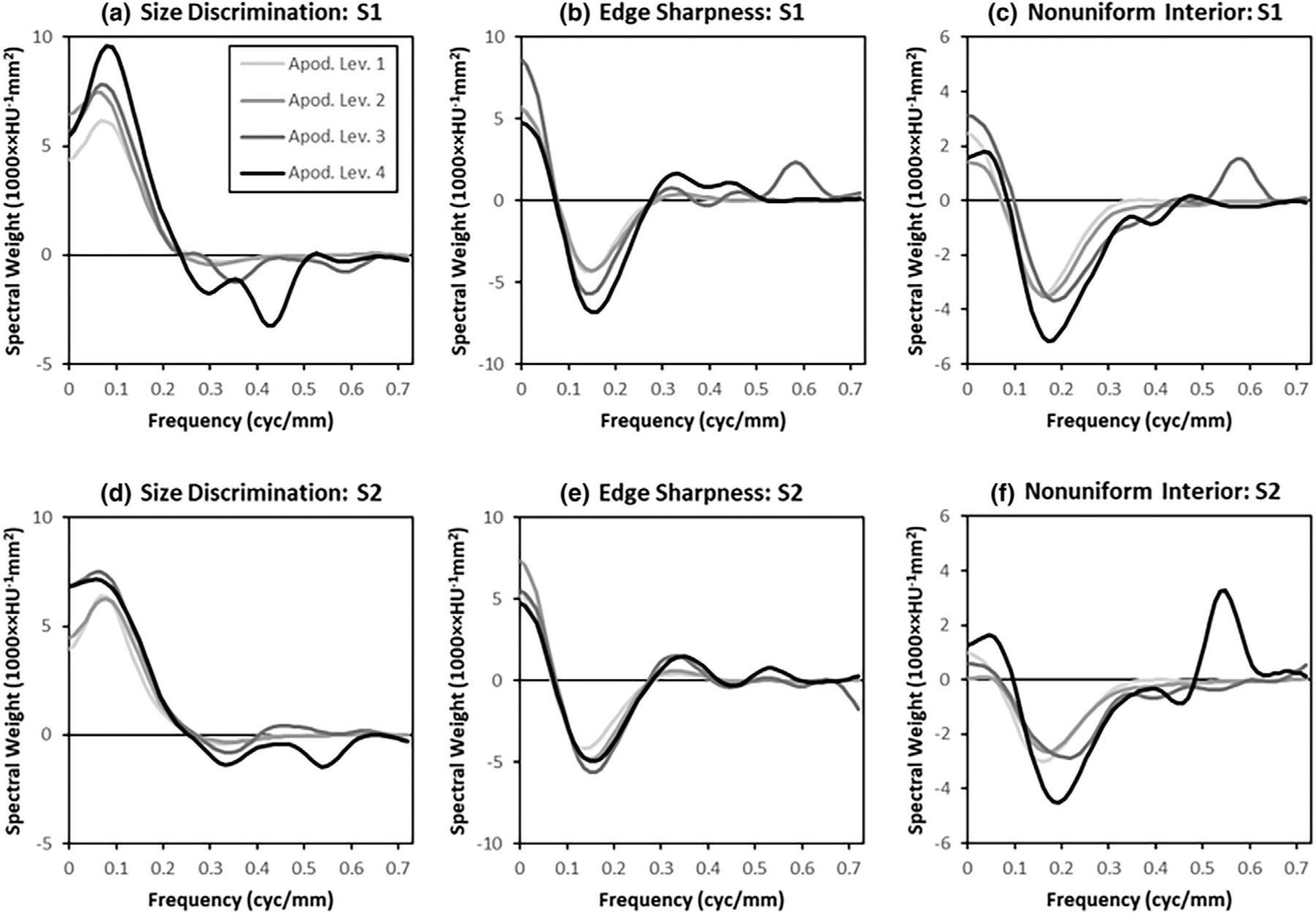
Classification image spectra. Average spatial-frequency weights of the classification images are plotted as a function of radial frequency (The legend in the upper left applies to all plots) for all apodization levels of each task and system resolution (S1: Low-resolution; S2: high-resolution). Each plot shows the radial average of the classification-image spectrum (averaged across subjects), which shows how subjects adapt to the different apodization conditions. In the highest levels of apodization (A3 and A4) the plots show some evidence instability at high frequencies (>0.35 cyc/mm) from inverting the noise covariance matrix.

The radial-frequency plots in Figure 7 show the frequency spectra of the classification images as described in Section 2.5.1. Each graph in Figure 7 shows all four apodization levels, which allows comparison of how apodization impacts the way observers access information in these tasks. To a greater or lesser extent, all of these plots indicate that as the amount of apodization is increased, spatial-frequency weights increase at higher spatial frequencies (>0.1 cyc/mm).

**FIGURE 8:**
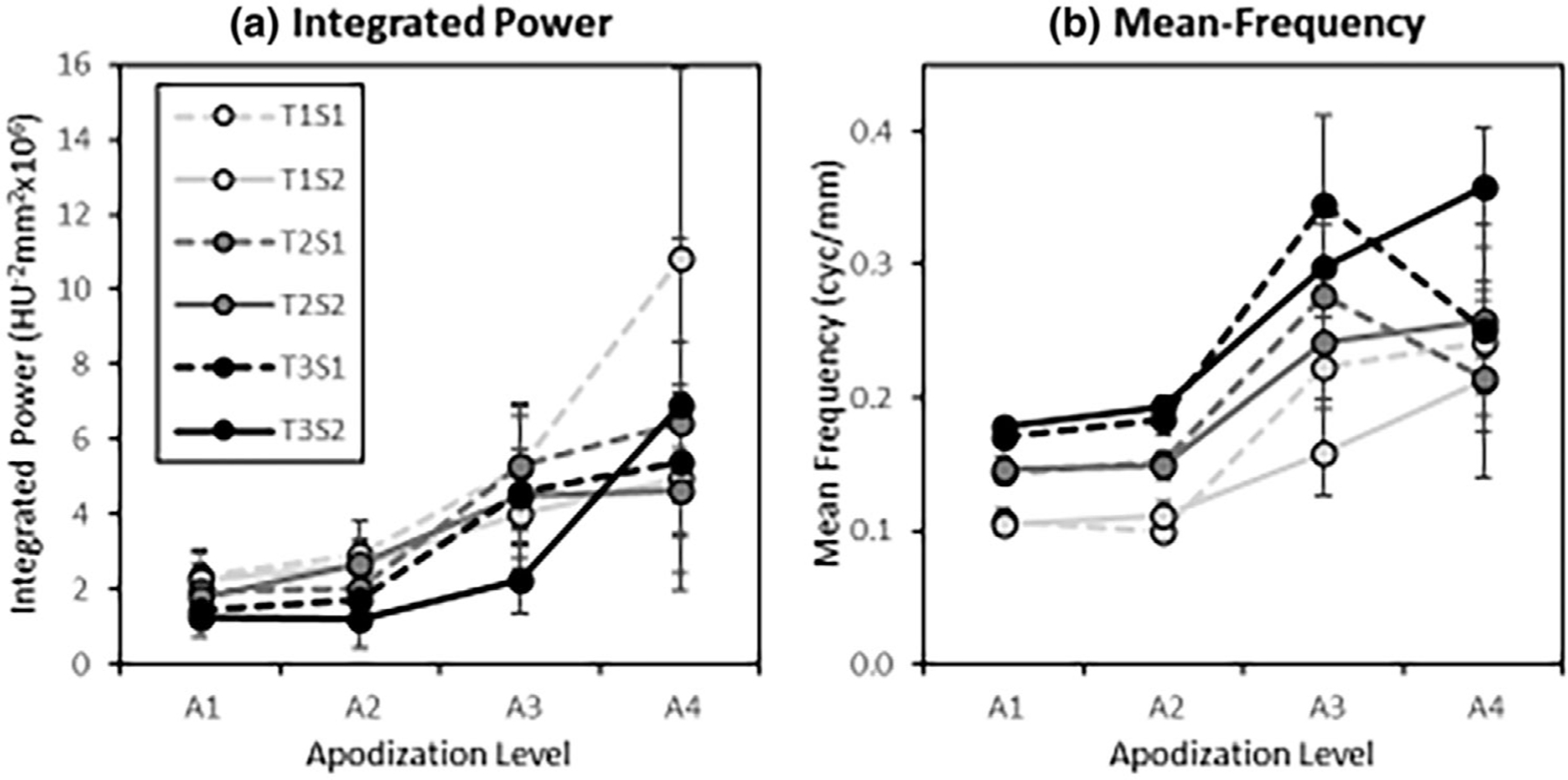
Classification-image feature values. The integrated power (a) and mean-frequency (b) features are plotted as a function of the apodization level (A1–A4) for each task (T1–T3) and imaging system (S1,S2). Each estimate is the average of the feature values, with error bars representing a 95% confidence interval on the mean across the six subjects in the studies. The legend applies to both plots.

The classification-image features plotted in Figure 8 provide quantitative support to these observations. These plots show the classification-image integrated power and mean frequency of the average classification images. Both features rise with apodization level, with a somewhat greater rise apparent for higher apodization levels in System 1 (low resolution) compared to System 2 (high resolution).

These differences may simply reflect the implementation of apodization in these studies, which has a stronger modulation for System 1 than System 2 (see Figure 1). Three-way ANOVA models have been fit for the Integrated-Power and Mean-Frequency features separately, with Task, System, and Apodization-Level as fixed main effects and all 2-way interactions. Subjects are modeled as random effects along with their interactions with each of the main effects.

For the Integrated-power feature, significant effects are found for main effects of task (*p* < 0.0005), system (*p* < 0.02), and apodization (*p* < 0.0001). There is also a significant interaction between Apodization and the imaging system (*p* < 0.019). For the mean-frequency feature, there are significant main effects of Task (*p* < 0.0001) and Apodization (*p* < 0.0003), and a significant interaction between System and Apodization (*p* < 0.002). These *p*-values are corrected for multiple comparisons using a 5% false discovery rate across both features.

### 3.4 | Sampling efficiency and differential sampling efficiency

**FIGURE 9:**
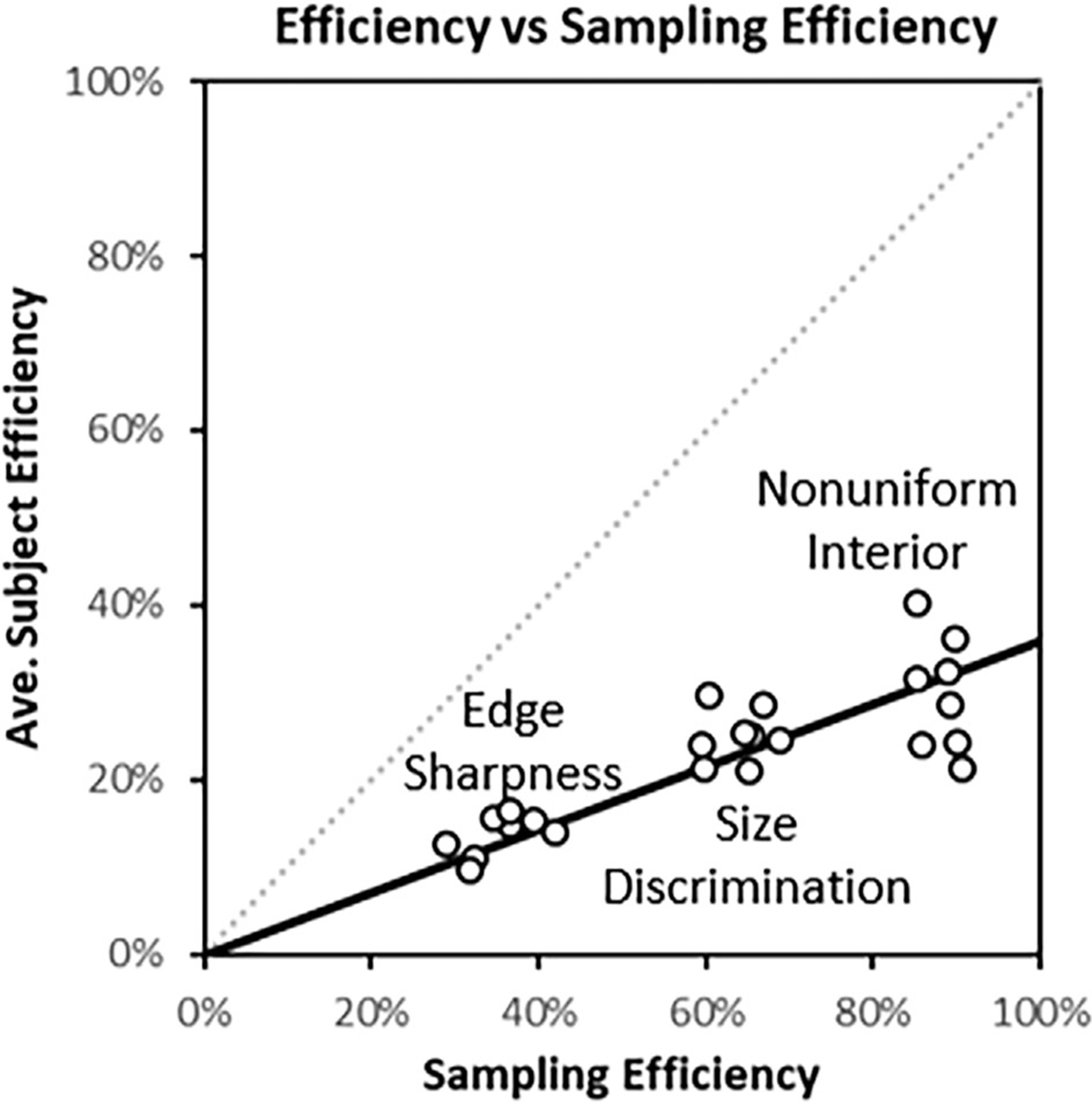
Sampling efficiency from classification images. Each symbol in the plot represents one of the 24 experimental conditions. The sampling efficiency of the classification images is associated with subject efficiency ($R^{2}=69.6\%$), and shows a clear distinction between the three different tasks (labels on plot).

Sampling efficiency of the classification images, described in Section 2.5.2, is compared to average subject efficiency through the scatterplot shown in Figure 9. Sampling efficiency is considerably higher than total efficiency, seen as a departure from the equivalence line in the plot. The best-fitting regression line has slope of 0.36, which indicates a substantial role for other sources of inefficiency, particularly internal noise. The association between efficiency and sampling efficiency is moderately high, with an $R^{2}$ value of 67.6%. The points also appear to cluster for each task, indicating that sampling efficiency is explaining task effects reasonably well. The $R^{2}$ value for average efficiency within each task is 93.7%. However, effects within a given task, such as apodization level, do not seem to be well explained by sampling efficiency.

**FIGURE 10:**
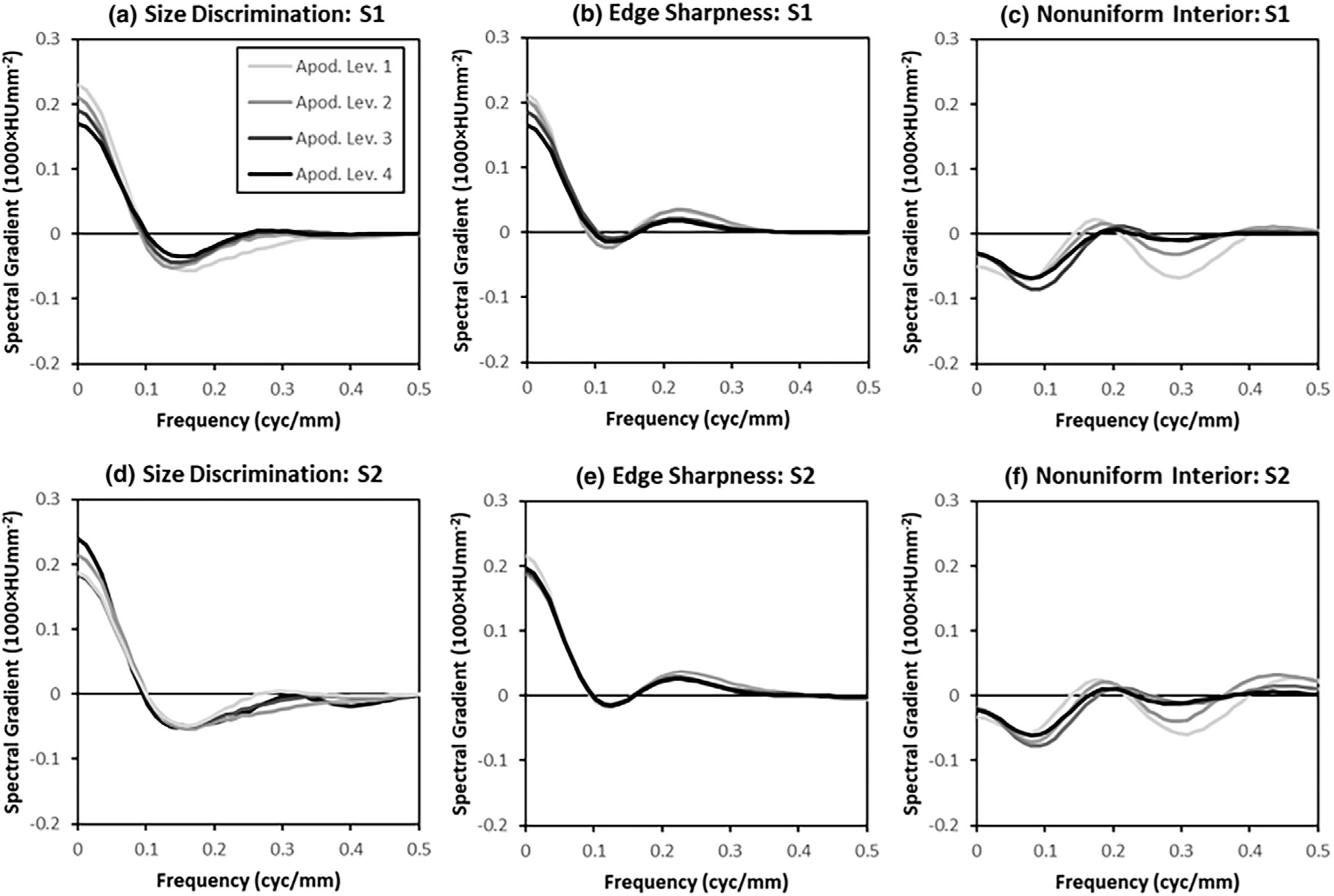
Differential-sampling-efficiency spectra. Radial averages of the differential sampling efficiency are plotted for the four apodization levels (Legend in upper left applies to all plots) within each task and system. The differential-sampling-efficiency spectra are derived from the classification-image spectra shown in Figure 7, and the show where the spectra are under-weighted (positive values) or over-weighted (negative values), as described in the text.

Plots of the differential sampling efficiency are shown in Figure 10, with the same grouping of results as the classification image spectra in Figure 7, from which they are derived. Recall from Section 2.5.3 that differential sampling efficiency values quantify the degree to which classification-image spectral weights are over- or underweighted. The plots show that for Tasks 1 and 2, there is substantial underweighting at low spatial frequencies. Interestingly, the lowest spatial frequencies appear to be over-weighted in Task 3, which may reflect low levels of signal in these frequencies (see Figure 1d). In the mid-range spatial frequencies (0.1–0.3 cyc/mm), the differential sampling efficiency appears to oscillate from underweighted to over-weighted with decreasing magnitude before decaying further at higher frequencies. This pattern of mismatch is consistent with subjects using an undersized discrimination template in the spatial domain. A spatially undersized template would expand and down-weight the classification image spectrum, leading to under-weighting the lowest spatial frequencies because of the down-weighting, and over-weighting higher frequencies as the frequency spectrum expands. Thus the differential sampling efficiency results may reflect some mismatch in the perceived size of the lesions.

### 3.5 | Unapodized classification images

**FIGURE 11:**
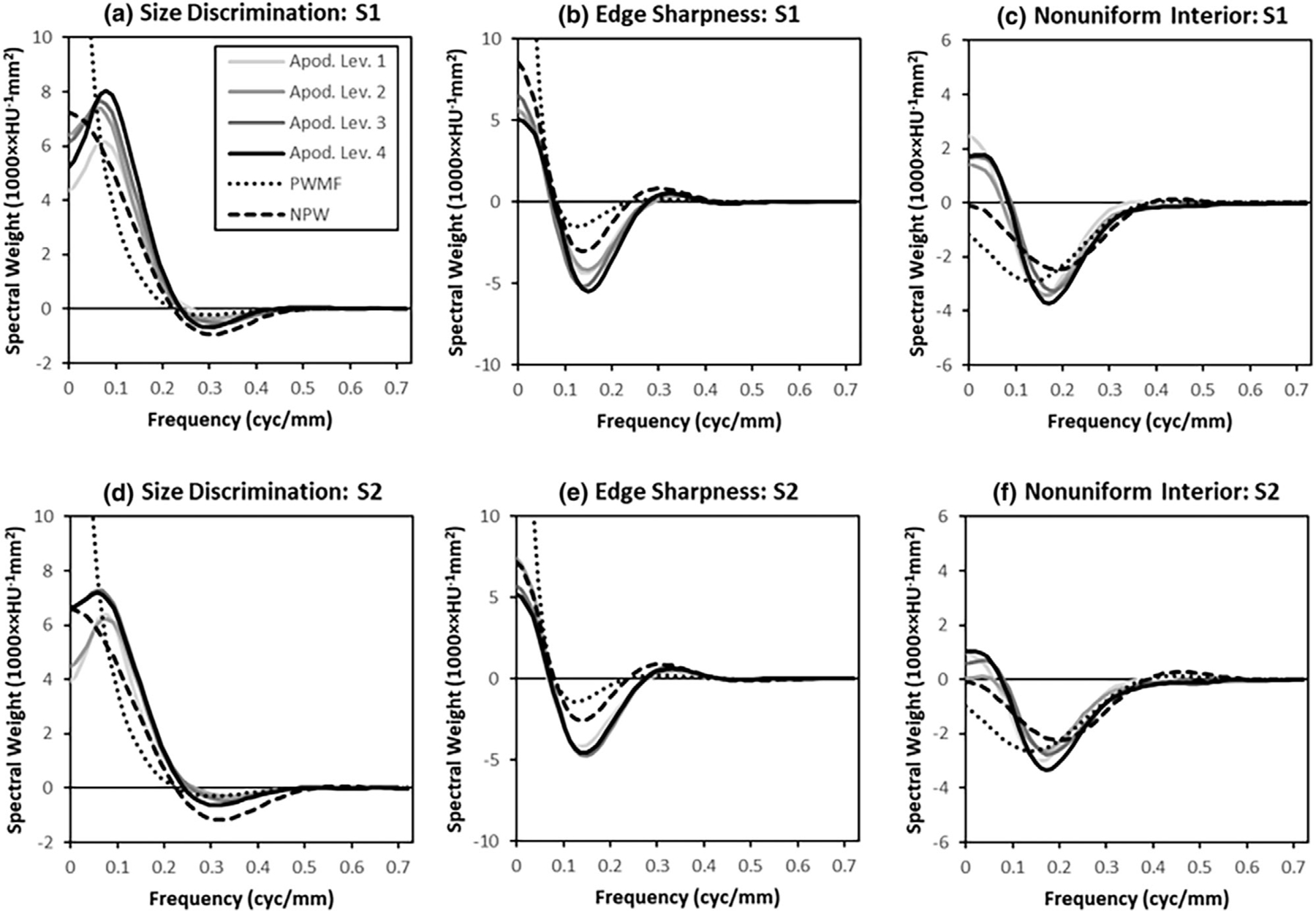
Unapodized classification image spectra. Average spatial-frequency weights of the unapodized classification images are plotted as a function of radial frequency. Each plot shows the average radial frequency weights for the classification image (averaged across subjects) using responses from each of the four apodization conditions (A1–A4), but constructed from unapodized noise fields (Legend in upper left applies to all plots). These plots show the impact of apodization on the spectral weights used by human observers. For comparison, we also plot the profile of the signal spectrum and the pre-whitened matched filter (PWMF), which represents optimal spatial weighting.

Figure 11 shows the unapodized classification image spectra plotted with the spectra of the difference signal and the prewhitened matched filter (PWMF) for comparison. The PWMF plots show the relatively large weights applied to low spatial frequencies by the ideal observer in Task 1 and 2. The lack of these weights in the observer classification images is a source of lower sampling efficiency in these tasks. Generally, the unapodized classification-image spectra in Figure 11 appear to be less variable than the standard classification-image spectra in Figure 7. This would suggest that observers are accounting for apodization to some extent as they perform the task under apodization conditions. These observations are assessed quantitatively using the classification-image features.

**FIGURE 12:**
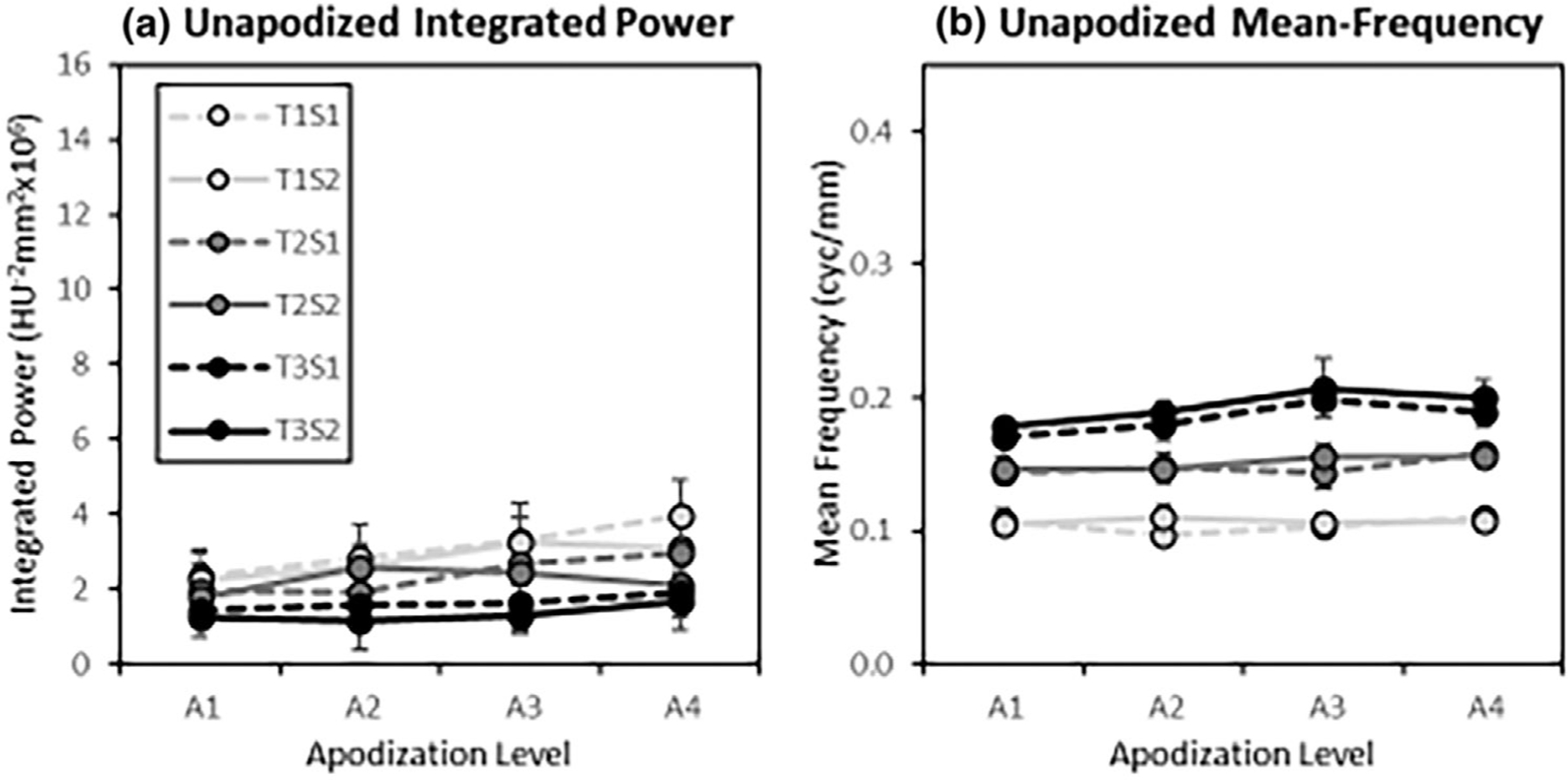
Unapodized classification image spectral features. The plots show the Integrated Power (a) and Mean Frequency (b) features plotted as a function of apodization level for the unapodized classification-image spectra. Plotted on the same scale as Figure 8 for comparison. While trends are similar to feature plots for the apodized classification images, the apodization effect is substantially reduced.

Integrated-power and mean-frequency features of the unapodized classification images are plotted in Figure 12. The feature values maintain the same relative ordering across task as was found in Figure 8, but there appears to be substantially less of an increase with apodization. In Figure 8, the integrated power is 257% higher in Apodization level 4 relative to Apodization level 1 on average. In Figure 12, this ratio is only 43% higher on average. The mean frequency values are 81% higher on average in Figure 8, and only 8% higher in Figure 12. While the trends in these spectral features are similar between apodized and unapodized classification images, the effect of apodization appears to have been substantially reduced. This means that the observers are adapting their discrimination procedure to a substantial degree in order to account for the apodization present in the images.

ANOVA modeling of the unapodized feature data find significant effects of both task and apodization for both integrated power (*p* < 0.0001 and *p* < 0.0009, respectively) and mean frequency features (*p* < 0.0001 and *p* < 0.0013, respectively) with false-discovery rate corrected *p*-values. However, while ANOVA effect sizes between the apodized and unapodized classification-image features are roughly constant for task effects, the apodization effect shrinks by a factor larger than 7. This provides additional evidence that apodization effects are reduced in the unapodized classification images, although they still appear to increase with apodization.

## 4 | DISCUSSION

The primary results described in Section 3 show that subjects exhibit performance effects across task and apodization level, with accompanying differences in the average subject classification images. Somewhat surprisingly, system resolution was not a significant factor in the efficiency and classification image results. This may be because the ramp-spectrum noise has increasingly high power at the higher spatial frequencies available in System 2, making the higher-resolution system (System 2) not particularly advantageous for these specific tasks. Further assessment will be needed to resolve this finding.

Two known components of visual performance are worth considering as mechanisms for low overall efficiency in these 3 discrimination tasks. The first of these is the inherent “lesion”contrast of the task, often referred to as the pedestal contrast. The second is the correlation structure of the noise. These mechanisms are discussed below in Sections 4.1 and 4.2. We are also interested in understanding the role of apodization in these tasks since apodization—or more generally image reconstruction—can be easily adjusted in practice to optimize task performance. Discussion of what the classification images reveal about apodization and subject performance is given in Section 4.3. And finally, some of the limitations of this study are given in Section 4.4.

### 4.1 | Contrast effects

The findings demonstrate that human observers are relatively inefficient in these featural discrimination tasks in ramp-spectrum noise. The low efficiency of human observers provides a rationale for using aids, such as digital calipers or radiomics algorithms to assist with the assessment of lesion features.^55^ In 1981, Burgess et al.^27^ established visual efficiency in noise at approximately 50%, with a range of 20%–70%. They used supra-threshold contrast discrimination tasks of Gaussian, Gabor-function, and 2-period sinusoid signals embedded in white noise. The efficiency results reported here are at the low end of his range, with the Task 2 results uniformly below the 20% lower limit described by Burgess et al. However, some subsequent studies have found lower efficiency values, particularly as contrast of the non-target profile (the “pedestal”) increases. For example, Kersten et al.^28^ looked at contrast discrimination tasks in noise for luminance disk profiles, with contrasts of the pedestals ranging from 5% to 43%. They found conditions with relatively low efficiency (∼10%), generally for the higher contrast pedestals.

Even though the tasks described here represent relatively low-contrast lesions by lung-cancer screening standards (∼200HU), we would suggest that this is relatively high-contrast in comparison to many discrimination studies in the vision literature. Peak lesion contrast of the displayed images is approximately 50% after the window and level settings are applied. This is higher than the highest pedestal contrast level in the Kersten study referred to above. The Burgess study, also referred to above, quantified the contrast of a Gaussian target (equivalent of the “benign lesion” in this work) with signal-energy to noise-spectral-density ratio (ENR) of 5. The ENR in this study ranges from 11.5 to over 1000 in the various conditions.

Low efficiency in discrimination tasks with high-contrast lesions is also consistent with previous findings of Abbey et al.,^49^ who compared efficiency in detection, contrast-discrimination, and identification tasks to show that human observers adapt their spatial weights to the pedestal of a task. The contrast-discrimination task in that work, which involved discriminating a contrast increment for a Difference-of -Gaussian (DOG) profile with a pedestal at 60% peak contrast, resulted in task efficiencies of 25% which are generally similar to the results found here. It is worth noting that the tasks used in this work are different than classical contrast-discrimination tasks in that the signal profile is not simply a scaled version of the pedestal profile. The tasks here evaluate fine features of a lesion boundary or of the lesion interior. Nonetheless, the efficiency results indicate that these tasks appear to follow the same general trends as classic contrast-discrimination tasks.

### 4.2 | Ramp-spectrum effects

Ramp-spectrum noise is also associated with low human-observer efficiency. Image texture with power that increases as a function of spatial frequency is the opposite of so-called “natural scenes” where power falls off, typically with an inverse square law.^56–59^ This leads to the plausible explanation that the human visual system is simply not well-adapted to ramp-spectrum noise.

The “ramp-spectrum” component of the noise rises approximately linearly at low spatial frequencies, even with apodization (see Figure 2). This means that that the lowest frequencies have relatively little noise, making any signal in these frequencies particularly informative. As shown in Figure 1, Tasks 1 and 2 have relatively high signal values at low spatial frequencies, with relatively little low-frequency signal for Task 3. Thus, the ideal observer will give these frequencies a relatively high weight when forming a decision variable. This can also be seen in the prewhitened matched filter plots in Figure 11, which heavily weight the lowest spatial frequencies for Tasks 1 and 2. The fact that human-observer efficiency is higher in Task 3, which has relatively little signal in the lowest frequencies, suggests that humans are not able to use these low spatial frequencies effectively.

This hypothesis is supported by the classification-image spectra in Figure 7, which do appear to have low frequency weights. However, the differential sampling efficiency plots in Figure 10 show that the low spatial frequencies are still relatively underweighted in Tasks 1 and 2, and this limits observer efficiency. One possible mechanism for this limited ability to use low-frequency information may be internal noise that has spectral component that matches natural scenes with a inverse-square power law. This would mean that low spatial frequencies have much more internal noise, and lead an observer to shift weights away from them. But further experiments will be needed to verify if this is how humans are working.

### 4.3 | Effect of apodization

The three effects investigated in this study (task, system, apodization) are controlled very differently in practice. The task is intended to represent the presentation of disease, which is not under the control of a practitioner. The imaging system is under the control of practitioners to some degree, although typically changing an imaging system for a new and improved device represents a substantial undertaking, making this a difficult factor to change. However, apodization is implemented directly through image reconstruction or in post-processing algorithms, which makes it comparatively easy to change and optimize.

Figures 7–9 give some indications of how observers respond to different levels of apodization on average, and generally show that the spatial frequency profile of the classification images changes across apodization conditions. However, these classification-image spectra are somewhat difficult to interpret because of changing signal and noise properties across apodization levels, and this motivates the analysis of unapodized classification images. The unapodized classification images show substantially less effect of an apodization effect. This means that observer weights are changing to make responses more invariant to apodization. Observers are using their ability to adapt to account for the apodization present in the images to some degree.

Nevertheless, the unapodized classification images still show an apodization effect of increased integrated power and mean frequency, although these effects are considerably attenuated relative to the standard classification images. So the net effect of apodization is to increase the spatial weights used in these experiments, particularly at higher spatial frequencies. This is a surprising finding, since the ostensible purpose of apodization is to suppress these higher frequencies, presumably to induce the observer to place more weight on lower frequencies. We find that after suppression of these frequencies by apodization filters, the observers appear to be effectively giving them more weight rather than less. Thus, quantitative features of the classification-image spectra support the idea that the net effect of frequency suppression by these apodization filters is an upweighting of higher spatial frequencies. This result bears further investigation to see if it holds generally.

### 4.4 | Study limitations

It is important to remain aware that our performance results are based on simulations of the imaging process with stylized object profiles and forced-choice tasks that are conducted in a laboratory environment with trained, but medically naïve, subjects. This abstraction of the imaging and image reading processes is useful for identifying and characterizing the mechanisms that may be operating in clinical settings, but they should not be interpreted as representing clinical performance, and they did not involve a thoracic radiologist in their design.

These experiments also investigate the discrimination of selected isolated features of lesions, and other effects may be present when the discrimination task involves different features or combinations of features to assess malignancy status, as is the case with clinical lesions in the lung. It is quite possible that more realistic lesions would emphasize high spatial frequencies even more strongly, and this may explain the lack of a clear effect for a higher MTF system in this study. Additionally, there are other sources of variability in imaging that can affect clinical performance (patient motion, beam-hardening artifacts, etc.) that are outside the scope of this study, which is focused on the specific impact of noise and noise texture.

## 5 | CONCLUSION

The lesion-discrimination tasks used in this study are representative of malignant features of lung lesions, and we find that they emphasize higher spatial frequencies than detection of the lesions themselves. This makes them a potentially useful means for assessing image-quality over a larger range of the frequency domain. Additionally, while observer efficiency varies across the three features tested (10%–40%), the low overall efficiency of human observers suggests that there may be considerable room for optimizing imaging methodology. For example, in these experiments image smoothing through high-frequency apodization was found to have a significant effect on both task performance and reader efficiency.

Classification-image analysis helps provide an explanation for the reader performance results, and points to the mechanisms underlying them. Estimates of sampling efficiency derived from the classification images explain the variability found across the three different tasks as variations in the tuning of the spatial templates used to perform them. The differential sampling efficiency, a concept introduced here, indicates that this is largely due to observers’ inability to adequately weight low spatial frequencies in ramp spectrum noise. Residual inefficiency is consistent with pedestal-masking effects that have been found widely in discrimination tasks.

However, improved performance and efficiency as a function of apodization are not explained by sampling efficiency in the way that effects of different discrimination features are. As the level of apodization increases, the classification images increase in terms of integrated power and mean frequency, indicating that observers are, to some extent, adapting to the level of apodization applied to the images. This explanation is supported by the unapodized classification images, which show markedly attenuated effects indicating that observer responses are relatively independent of apodization filtering. The effects of apodization in this analysis are consistent with a reduction of internal noise that increases the integrated power of a classification image.

Taken in whole, the results reported here provide additional evidence that human observers are able to partially adapt to different statistical properties in simple tasks. This information helps provide a better understanding of how human observers use the information in CT images, and should assist in the development of more accurate models of discrimination performance for the optimization of medical imaging systems.

## Figures and Tables

**TABLE 1:** Task descriptions. The three tasks used in for this work are listed (with abbreviations) along with the base lesion diameter.

Label	Feature description	Base diameter
Task 1 (T1)	Size discrimination	3 mm
Task 2 (T2)	Edge sharpness	5 mm
Task 3 (T3)	Nonuniform interior	5 mm

**TABLE 2:** Resolution, and noise. The effect of apodization on resolution (MTF = 10% of max) and noise (pixel Std. Dev.).

	Res. (Cyc/mm)		Noise SD (HU)	
Apod.	S1	S2	S1	S2
A1	0.47	0.58	166.5	166.5
A2	0.45	0.56	112.9	129.9
A3	0.39	0.49	46.5	65.0
A4	0.34	0.43	30.2	42.2
